# BET degraders reveal BRD4 disruption of 7SK and P-TEFb is critical for effective reactivation of latent HIV in CD4+ T-cells

**DOI:** 10.1128/jvi.01777-24

**Published:** 2025-03-11

**Authors:** Anne-Marie W. Turner, Frances M. Bashore, Shane D. Falcinelli, Joshua A. Fox, Alana L. Keller, Anthony D. Fenton, Renee F. Geyer, Brigitte Allard, Jennifer L. Kirchherr, Nancie M. Archin, Lindsey I. James, David M. Margolis

**Affiliations:** 1 UNC HIV Cure Center, University of North Carolina 2331 , Chapel Hill, North Carolina, USA; 2 Division of Infectious Diseases, Department of Medicine, University of North Carolina School of Medicine, University of North Carolina 6797 , Chapel Hill, North Carolina, USA; 3 Center for Integrative Chemical Biology and Drug Discovery, Division of Chemical Biology and Medicinal Chemistry, UNC Eshelman School of Pharmacy, University of North Carolina at Chapel Hill 15521 , Chapel Hill, North Carolina, USA; 4 Department of Microbiology and Immunology, University of North Carolina School of Medicine, University of North Carolina at Chapel Hill 2331 , Chapel Hill, North Carolina, USA; 5 Lineberger Comprehensive Cancer Center, School of Medicine, University of North Carolina at Chapel Hill 169113 , Chapel Hill, North Carolina, USA; Icahn School of Medicine at Mount Sinai, New York, New York, USA

**Keywords:** BRD4, chemical degraders, HIV, latency reversal, P-TEFb

## Abstract

HIV cure strategies that aim to induce viral reactivation for immune clearance leverage latency reversal agents to modulate host pathways which directly or indirectly facilitate viral reactivation. Inhibition of bromo and extra-terminal domain (BET) family member BRD4 reverses HIV latency, but enthusiasm for the use of BET inhibitors in HIV cure studies is tempered by concerns over inhibition of other BET family members and dose-limiting toxicities in oncology trials. Here, we evaluated the potential for bivalent chemical degraders targeted to the BET family as alternative latency reversal agents. We observed that despite highly potent and selective BRD4 degradation in primary CD4+ T-cells from ART-suppressed donors, BRD4 degraders failed to induce latency reversal as compared to BET inhibitors. Furthermore, BRD4 degraders failed to mimic previously observed synergistic HIV reactivation between BET inhibitors and an activator of the non-canonical NF-κB pathway. Mechanistic investigation of this discrepancy revealed that latency reversal by BET inhibitors is not related to the abatement of competition between Tat and BRD4 for P-TEFb, but rather the ability of BRD4 to disrupt 7SK and increase the levels of free P-TEFb. This activity is dependent on the shift of BRD4 from chromatin-bound to soluble and retargeting of P-TEFb to chromatin, which is dependent on intact BRD4 but independent of the bromodomains.

**IMPORTANCE:**

Multiple factors and pathways contribute to the maintenance of HIV latency, including bromo and extra-terminal domain (BET) family member BRD4. While small molecule inhibitors of the BET family result in latency reversal, enthusiasm for the use of BET inhibitors in HIV cure is limited due to toxicity concerns. We examined BRD4-selective chemical degraders as alternatives to BET inhibitors but found two robust degraders failed to induce latency reversal. We observed key differences in the ability of BET inhibitors versus BET degraders to disrupt P-TEFb, a key cellular activator of transcription and a complex required for HIV reactivation. We present a new model for the role of BRD4 in HIV latency and propose that BRD4 be reconsidered as an activator rather than a repressor of HIV transcription in the context of HIV cure strategies.

## INTRODUCTION

Advances in antiretroviral therapy have made human immunodeficiency virus (HIV) a chronic but manageable disease. However, a cure remains elusive due to a persistent, transcriptionally repressed latent reservoir in resting CD4+ T-cells (rCD4). One approach to an HIV cure focuses on latency reversal and clearance. In a two-component system, latency reversal is dependent on the reactivation of the latent virus reservoir followed by targeted clearance of reactivated cells using methods that boost recognition by the immune system (1). Current latency reversal agents (LRAs) rely on disruption or modulation of host pathways which directly or indirectly result in viral reactivation; however, to date, none have proven successful in reducing the size of the latent reservoir (1). As such, there is a constant need to identify and evaluate new small molecules targeting well-defined HIV latency pathways for improved specificity and activity. This approach may increase the safety and efficacy of cure studies.

The bromo and extra-terminal domain (BET) family includes ubiquitously expressed BRD2, BRD3, BRD4, and tissue-restricted BRDT. All family members contain tandem bromodomains (BD) which bind acetylated substrates, primarily histone tails, and an extra-terminal (ET) domain involved in additional protein-protein interactions (2, 3). BRD2/3/4 are recognized global regulators of transcription, with BRD4 being the most studied. BRD4 has three isoforms, the long isoform BRD4L and two shorter isoforms BRD4Sa and BRD4Sb which differ by a short unique C-terminal sequence on the latter (2, 3). BRD4L contains a unique C-terminal domain (CTD) that interacts with positive transcription elongation factor b (P-TEFb) (2, 3). P-TEFb, a heterodimer of CyclinT1/T2 and kinase CDK9, is critical for the effective release and elongation of paused RNA polymerase II (RNAPII) (4). Recruitment of P-TEFb to paused RNAPII by BRD4, the super elongation complex (SEC), or, in the case of HIV, the viral protein Tat, results in CDK9-mediated phosphorylation of the CTD of RNAPII, DSIF, and NELF, allowing for productive transcriptional elongation (4).

Early studies of the role of BET proteins in HIV focused on the BRD4/P-TEFb axis, demonstrating that BRD4 recruitment of P-TEFb could activate reporters containing the HIV promoter (LTR, long terminal repeat) in Tat-free systems (5). However, overexpression of Tat appeared to compete against this activity (6). Later work observed that overexpression of a peptide fragment containing amino acids 1209-1362 of BRD4, termed the PID (P-TEFb interacting domain), inhibited Tat-transactivation of the HIV LTR and association with CDK9, suggesting BRD4 and Tat competed for P-TEFb (7). The development of one of the first bromodomain inhibitors (BETi) JQ1 in 2010 (8) provided the first demonstration that bromodomain inhibition could reverse HIV latency (9–12). These works resulted in the prevailing model that BRD4 bound at the HIV promoter acts to repress HIV transcription by blocking the Tat/P-TEFb interaction and that inhibition and disruption of BRD4 from chromatin relieve this competition. Two groups further described the ability of JQ1 to disrupt P-TEFb from the repressive non-coding ribonucleoprotein (RNP) complex 7SK, providing an additional pathway that could contribute to both Tat-dependent and independent activation (10, 11). Further work on BRD4 restriction of HIV has implicated the short isoforms of BRD4 (BRD4S), which lack the PID, in the recruitment of the repressive chromatin remodeling BAF complex to the LTR (13) and the maintenance of repressive BRD4 at the LTR via KAT5-mediated histone 4 acetylation (H4ac) (14, 15). Studies using shRNA or CRISPR knockdown of BRD2 suggest some involvement in latency reversal; however, whether this is a direct or secondary impact of BRD2 depletion and the potential mechanisms are currently unknown (16, 17).

**Fig 1:**
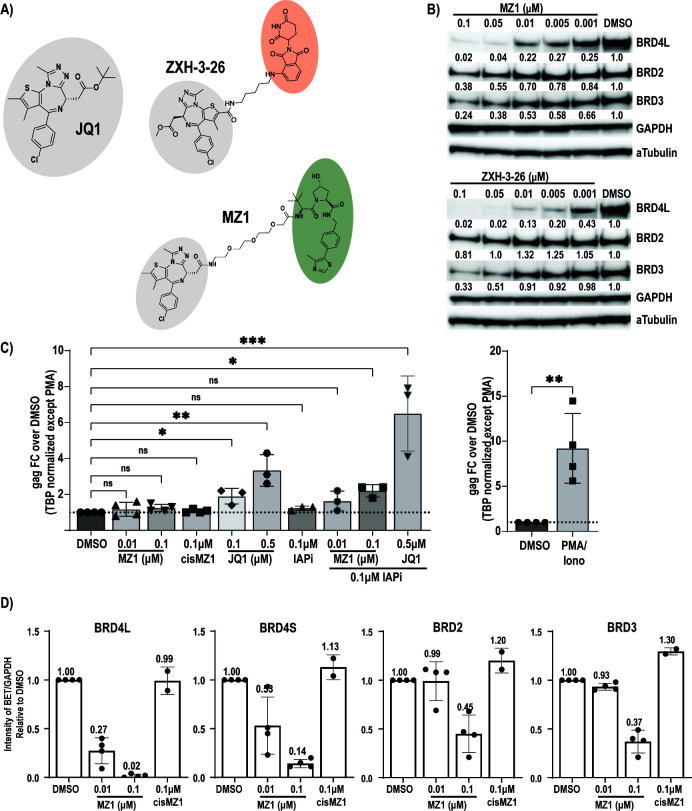
HIV latency reversal in ART-suppressed donors by bivalent degraders of the BET proteins. (**A**) BET inhibitor JQ1 is highlighted in gray. Degraders MZ1 and ZXH-3-26 use JQ1 linked to either Von Hippel-Lindau (VHL) (green) or CRBN (orange) recruiting ligands. (**B**) A targeted dose titration of MZ1 and ZXH-3-26 in healthy, primary CD4+ T-cells (*n* = 1 donor) identifies BRD4-specific degradation at 10 nM and 5 nM, respectively. Relative protein levels were determined by standardizing each protein to the GAPDH loading control, followed by expression relative to the dimethyl sulfoxide (DMSO) control. (**C**) Total CD4+ T-cells from ART-suppressed donors were treated for 24 hours with MZ1 alone (*n* = 4, SD) or in combination with Inhibitor of Apoptosis inhibitor (IAPi) AZD5582 (*n* = 3, SD) and induction of gag cell-associated RNA assayed by quantitative real-time PCR. Four to five replicates of 1–2E6 cells per donor per condition were assayed. Each dot represents the average of these replicates per unique donor. Phorbol 12-myristate 13-acetate (PMA)/Ionomycin results are graphed independently due to differences in normalization (see Materials and Methods). Significance was assessed relative to DMSO control by Kruskal-Wallis with uncorrected Dunn’s multiple comparison test, (*) *P* < 0.05, (**) *P* < 0.01, (***) *P* < 0.001, (****) *P* < 0.0001. (**D**) BET protein levels as assayed by western blot in the 4 ART-suppressed donors (*n* = 4, SD). Cells were treated concurrently with those for RNA isolation. BET protein levels are quantitated relative to GAPDH levels for all donors. cisMZ1 could not be assayed in all donors due to cell limitations (*n* = 2, range).

BRD4 inhibition remains an attractive target for latency reversal, but enthusiasm for the use of BETi in HIV cure studies is tempered by the fact that current BETi are pan-inhibitors, and data from oncology studies have demonstrated unfavorable toxicities including thrombocytopenia and severe gastrointestinal symptoms (18, 19). A new class of molecules, proteolysis-targeting chimeras (PROTACs), has recently emerged as a potential alternative to BETi. PROTACs, also referred to as bivalent chemical degraders, aim to degrade the protein of interest rather than simply inhibit its function. Specifically, these degraders are a bifunctional molecule that simultaneously binds an E3 ubiquitin ligase and a protein of interest (POI), resulting in ubiquitination of the POI by the E3 ligase complex and POI degradation by the proteasome (Fig. 1A). Unique features of chemical degraders include (i) degradation versus inhibition of target molecules, (ii) ability to use POI-targeting ligands that do not bind the active site, (iii) the catalytic nature of the reagent which allows use at sub-stoichiometric concentrations, and (iv) the design of the bivalent molecule can result in improved selectivity over the POI-targeting ligand alone (20, 21). Thus, there is considerable interest in developing degraders for clinical applications (22). Furthermore, multiple degraders have been described with BRD4-selective degradation profiles (23–25).

To evaluate the potential for BET degraders as LRAs, multiple compounds were evaluated for target specificity and HIV latency reversal in both Jurkat-derived models and in CD4+ T-cells from ART-suppressed donors. Interestingly, despite the identification of two BET degraders with BRD4 selectivity, highly potent BRD4 degradation failed to induce latency reversal in cells from ART-suppressed donors as compared to BETi. Furthermore, BRD4 degraders failed to mimic synergistic HIV reactivation previously observed between BETi and an activator of the non-canonical NF-κB pathway (16). We endeavored to understand this discrepancy and found that the primary mechanism of HIV latency reversal by BETi is not the removal of BRD4 from chromatin but the disruption of P-TEFb from the repressive 7SK non-coding RNA complex. Of note, this activity was dependent on the physical BRD4 protein but independent of the bromodomains. These results further emphasize P-TEFb restriction in CD4+ T-cells as a main driver in the maintenance of HIV latency.

## RESULTS

### Selective BRD4 degradation does not induce latency reversal in HIV+ CD4+ T-cells

We recently published that activation of the non-canonical NF-κB pathway using the Inhibitor of Apoptosis inhibitor (IAPi) AZD5582 synergizes with BET inhibitors to strongly induce HIV *gag* cell-associated RNA in cells from aviremic donors (16). In Falcinelli et al., we also examined the ability of the chemical degrader ZXH-3-26 to induce gag cell-associated RNA alone and in combination with AZD5582 (16). Unexpectedly, we observed little to no induction of HIV *gag* cell-associated RNA with ZXH-3-26 alone despite robust BRD4 degradation at 0.005 µM and no evidence of synergy with AZD5582 (16). With consideration of the canonical model of the role of BRD4 in the maintenance of HIV latency, we sought to reproduce this result and further examine why targeted degradation of BRD4 did not mimic the latency reversal observed with BET inhibitors.

Highly BRD4 selective ZXH-3-26 uses BETi JQ1 as the targeting moiety and recruits the cereblon (CRBN) E3 ligase complex (24) (Fig. 1A). The degrader MZ1 also uses JQ1 but recruits the Von Hippel-Lindau (VHL) complex and was described as BRD4 selective below 0.25 µM (25). *cis*MZ1, a diastereomer of MZ1 containing a *cis*-hydroxyproline within the VHL ligand that abrogates VHL binding, was used as a control. To determine effective concentrations at which we could reproducibly and selectively degrade BRD4, we treated primary CD4+ T-cells with a broad concentration range for 24 hours and assayed BRD4L/S, BRD2, and BRD3 levels by western (Fig. S1A and B). We targeted <0.1 µM for further testing and observed no impact on protein levels using controls JQ1 and cisMZ1 (Fig. S1C and D). A targeted dose titration ranging from 0.001 µM to 0.1 µM of MZ1 and ZXH-3-26 demonstrated selective degradation of BRD4 at 0.01 µM by MZ1 and 0.005 µM by ZXH-3-26 (Fig. 1B). For both degraders, concentrations ≥0.05 µM demonstrated some reduction of BRD2 and BRD3 protein levels (Fig. 1B). To examine the speed of degradation, we performed an 8 hour timecourse in primary CD4+ T-cells. At 8 hours, 0.05 µM ZXH-3-26 degraded over 90% of BRD4L while 0.005 µM ZXH-3-26 and 0.1 µM MZ1 exhibited approximately 70%–75% reductions in BRD4L (Fig. S1E through H). A 0.01 µM MZ1 displayed the slowest degradation with only 30% lost after 8 hours (Fig. S1G). To focus on the primary effect of BRD4 degradation, we focused on a 24 hour timepoint for all future experiments.

We next repeated latency reversal experiments using MZ1 in primary CD4+ T-cells of ART-suppressed donors to match our prior ZXH-3-26 studies. At 0.01 µM (BRD4-selective) and 0.1 µM (pan-degradation), MZ1 alone failed to induce HIV *gag* cell-associated RNA as compared to JQ1 (Fig. 1C). Like ZXH-3-26, 0.01 µM MZ1 showed no synergy with AZD5582 as compared to JQ1/AZD5582 (Fig. 1C), despite specifically decreasing levels of both the BRD4L and BRD4S isoforms while sparing BRD2 and BRD3 (Fig. 1D). Individual western blots for each donor are provided in Fig. S2A and D. There was minimal impact on cellular viability by MZ1 alone and in combination with AZD5582 via measurement of total cellular ATP levels (Fig. S2E). In addition to our MZ1 data, we provide westerns characterizing ZXH-3-26 degradation in two donors (Fig. S3A and B) not previously published and provide calculations of average degradation efficacy for all BET proteins in the three donors assayed in Falcinelli et al. (Fig. S3C through F). Collectively, BRD4-selective and pan-BET degradation by two potent bivalent degraders failed to reactivate HIV, both alone and in combination with IAPi, implicating a non-bromodomain-related mechanism for HIV latency reversal in primary CD4+ T-cells and the requirement of the physical BRD4 protein.

### BRD4-selective degradation by BET degraders fails to induce latency reversal in Jurkat-derived latency models

**Fig 2:**
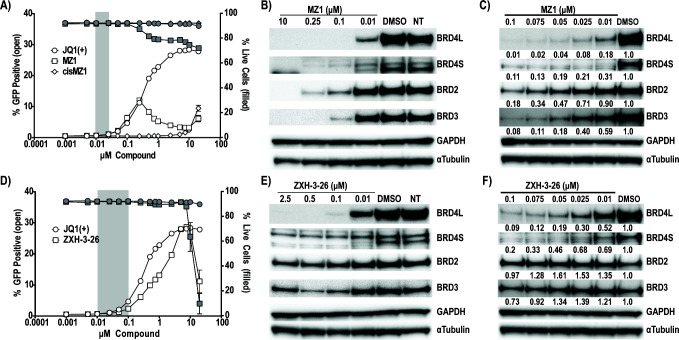
Latency reversal and targeted degradation of BET degraders in a Jurkat-derived Latency Model. Latency reversal was assessed via the treatment of JLatA2 cells with a 16-point dose titration of (**A**) MZ1 and *cis*MZ1. JQ1(+) data is overlayed and provided in Fig. S4. Each titration was performed two independent times with biological triplicates (*n* = 6, SEM) with GFP (open symbols) assessed by flow cytometry as a measure of latency reactivation and viability by live/dead stain (gray symbols). Error bars not extending past the symbol are not visible. The window of BRD4-specific degradation for MZ1 is highlighted in gray as determined by corresponding westerns for both BRD4L(ong) and BRD4S(hort) isoforms as well as BRD2 and BRD3 in JLatA2 cells (**B and C**). Matching experiments were performed for (**D**) ZXH-3-26 and the window of BRD4-selective degradation is highlighted in gray based on corresponding westerns (**E and F**). Relative protein levels were determined by standardizing each protein to the GAPDH loading control, followed by expression relative to the DMSO control. Western blots are representative of at least two independent experiments (also see Fig. 4B; Fig. S6B and C).

Knockdown of BRD4 using shRNA/siRNA approaches has been demonstrated to induce latency reversal in Jurkat-derived models of HIV latency (11, 17). We next performed a 16-point dose titration of MZ1 and ZXH-3-26 in the Jurkat-derived JLatA2 reporter line. JLatA2 cells contain an LTR-Tat-IRES-GFP reporter in which LTR activation drives GFP expression (26) and have previously been used in the study of BRD4 in HIV latency (11, 17). JQ1(+) and stereoisomer negative control JQ1(−) (8) were used to compare degrader activity to the parent BETi molecule. Latency reversal by JQ1(+) is graphed in the open circles in all graphs to allow direct comparison. As expected, JQ1(+) induced latency reversal as measured by the percent GFP positive JLatA2 cells by flow cytometry, with maximal activation inducing approximately 30% of the population (Fig. 2A and D, open circles). JQ1(−) showed no latency reversal (Fig. S4A, open squares). Neither compound showed a measurable impact on cellular viability as measured by a fixable live/dead stain (Fig. S4A, dark gray filled symbols). JQ1(+) treatment did not induce any changes in the protein level of any BET family members (Fig. S4B and C).

While both MZ1 and ZXH-3-26 demonstrated latency reversal in JLatA2 cells as measured by GFP induction (Fig. 2A and D), this occurred outside the range of BRD4-selective degradation as assayed by western blot using both extended and targeted concentrations for both degraders (Fig. 2B through E and F). The BRD4-selective range for both degraders is highlighted in gray in Fig. 2A and D. Statistical analysis using the Kruskal-Wallis Test with uncorrected Dunn’s multiple comparisons demonstrated no significant upregulation of GFP at BRD4-selective concentrations (Table S1). All assays were performed at 24 hours, suggesting HIV latency reversal is not a primary consequence of BRD4 degradation.

MZ1 displayed a unique pattern whereby GFP levels initially increased but then reached a concentration at which both GFP and cellular viability began to decrease (Fig. 2A). The concentrations at which this occurred correlated with near complete degradation of all BET family members as measured by western blot (Fig. 2B). These observations are consistent with previously published work by Winters et al. which observed significant defects in transcriptional initiation and elongation when using the BET degrader dBET6 at pan-degrading concentrations (27).

We observed some indication of the hook effect, the concentration at which the bivalent molecule is saturating such that the ternary complex is not formed and the individual parent inhibitors can act independently (20, 21), by both degraders. Both MZ1 (Fig. 2A, open squares) and *cis*MZ1 (Fig. 2A, open diamonds) at concentrations ≥10 µM induced GFP expression in JLatA2 cells. Poor cellular permeability due to the high molecular weight of chemical degraders has been characterized, with a recent study demonstrating that MZ1 is over 165,000-fold less permeable than JQ1 (28). GFP induction by *cis*MZ1 at 10 µM suggests the intracellular concentration of *cis*MZ1 approaches concentrations at which the JQ1 moiety alone can induce latency reversal. However, we did not observe a return of protein levels at 10 µM by western at the 24 hour time point. We observed recovery of BRD2 and BRD3 protein levels in ZXH-3-26 treated cells at 2.5 µM, but BRD4 protein levels did not show recovery (Fig. 2E). Coupled with our MZ1 observations, it may be that the recovery of BRD4 is either under our limit of detection by western or BRD4 recovery differs from BRD2/3 depending on the selectivity of the degrader. However, for both degraders, this occurred at significantly higher concentrations than those at which BRD4-selective degradation occurred and at which we assayed in latency reversal and thus had no relevance to the lack of observed induction of HIV.

### Viral reporter contributes to perceived latency reversal and IAPi synergy in Jurkats

**Fig 3:**
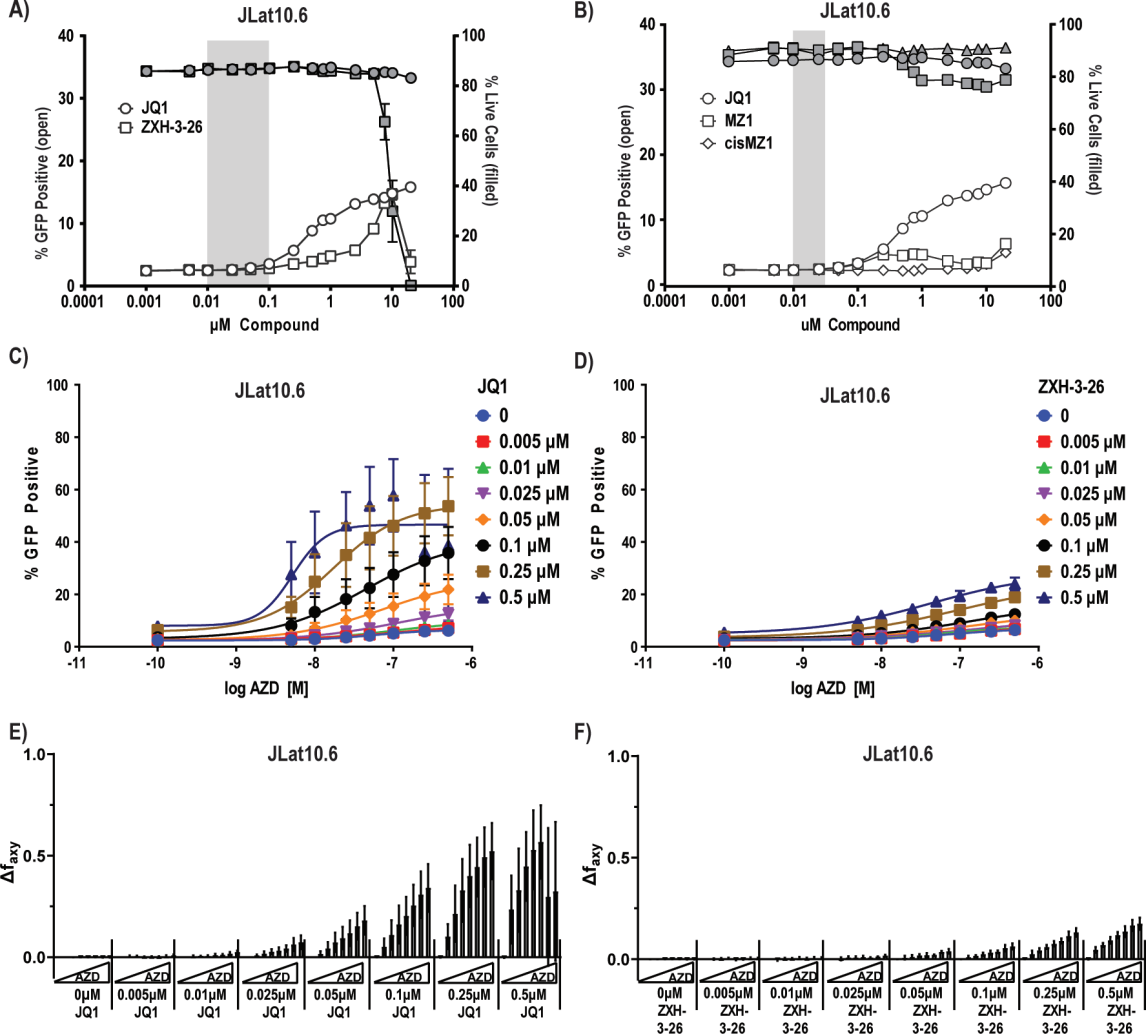
Latency reversal and synergy with AZD5582 by degrader ZXH-3-26 is attenuated in a full-length Jurkat cell line. A 16-point dose titration of (**A**) ZXH-3-26 and JQ1 or (**B**) MZ1 and JQ1 in JLat10.6 cells. Each titration was performed two independent times with biological triplicates (*n* = 6, SEM). Error bars not extending past the symbol are not visible. (**C**) 8-point dose titration of AZD-5582 and JQ1 in JLat10.6 cells. (**D**) 8-point dose titration of AZD-5582 and ZXH-3-26 in JLat10.6 cells. Data represents three independent experiments of each 8-point titration (*n* = 3, SD). Bliss synergy calculations of (**E**) AZD5582/JQ1 and (**F**) AZD5582/ZXH-3-26 in JLat10.6 cells based on data in C/D (*n* = 3, SD). Synergy experiments in JLatA2 cells are provided in Fig. S5.

While treatment of JLatA2 cells with MZ1 and ZXH-3-26 did not demonstrate latency reversal at BRD4 selective concentrations, we observed induction of GFP at higher, non-selective concentrations (Fig. 2). We sought to determine if this was unique to the JLatA2 line as different reporter lines are known to demonstrate variable latency reversal in response to the same LRA (29). As previously described, JLatA2 cells contain a short, 1.5 kB reporter. JLat10.6 cells contain a defective but near full-length viral reporter containing a frameshift in *env* and GFP in the place of *nef* (26, 30). When treated with the cytokine tumor necrosis factor alpha (TNFα), JLat10.6 cells reactivate to similar levels as JLatA2 cells (Fig. S5A and B). We performed the same 16-point titration of MZ1, *cis*MZ1, ZXH-3-26, and JQ1 in JLat10.6 cells. While JQ1 latency reversal was also attenuated as compared to JLatA2 cells, both ZXH-26 and MZ1 failed to induce notable latency reversal in JLat10.6 cells (Fig. 3A and B).

We next examined the impact of the reporter line on our observations of synergy between BETi, BET degraders, and IAPi. We performed an 8-point cross titration between AZD5582 and either JQ1 or ZXH-3-26 in both cell lines and used the Bliss Independence Model to analyze whether the combinations displayed antagonism (Δf_axy_ < 0), independence (Δf_axy_ = 0), or synergy (Δf_axy_ > 0). Consistent with our previous observations, AZD5582 in combination with JQ1 induced synergy in both cell lines (Fig. 3C and Fig. S5C). Interestingly, ZXH-3-26 in combination with AZD5582 in JLatA2 cells resulted in observable synergy even at BRD4-selective concentrations (Fig. S5D) while no synergy was observable at the same concentrations in JLat10.6 cells (Fig. 3D). Calculations of Bliss synergy for cross titrations are provided in Fig. 3E and F for the JLat10.6 line and Fig. S5E and F for the JLatA2 line.

The lack of observable synergy in JLat10.6 cells was consistent with our prior results in Falcinelli et al. (16) using another full-length HIV reporter cell line model. We hypothesize the smaller reporter virus construct in the JLatA2 reporter line has a lower barrier to productive transcriptional elongation and is more susceptible to secondary cellular effects of LRAs. Taken together with the observation that BRD4-selective concentrations fail to induce latency reversal in a broad dose-response curve, we find rapid and robust BRD4 degradation by small molecule degraders does not induce HIV latency reversal in primary cells or Jurkat reporter models.

### Modulation of P-TEFb/7SK differs between BRD4 degradation and inhibition

Three proposed mechanisms for the latency reversal activity of BETi are (i) reduction of competition for P-TEFb between Tat and BRD4, (ii) release of P-TEFb from the 7SK RNP, and (iii) maintenance of repressive BAF at the LTR by BRD4S (9–13). We demonstrated that both MZ1 and ZXH-3-26 degrade the BRD4S isoform with no impact on latency reversal (Fig. 1 and 2). To further understand how BETi but not BET degraders reverse HIV latency, we performed immunoprecipitations of CyclinT1 to identify levels of interacting BRD4 to assess competition for available P-TEFb and examined HEXIM upregulation as a proxy for P-TEFb release. The 7SK RNP is composed of the 331 nucleotide non-coding RNA 7SK and major proteins LARP7, MeCBP, and HEXIM (4). HEXIM (Hexamethylene-bis-acetamide Inducible Transcript 1) directly interacts with stem loop 1 (SL1) of 7SK and with the P-TEFb heterodimer, blocking the ATP binding pocket of CDK9 and sequestering P-TEFb in this repressive RNP complex (31, 32). Disruptions of the 7SK/P-TEFb equilibrium by cellular stress, stimuli, or small molecules have been previously shown to induce a feedback loop in which the release of excess P-TEFb triggers upregulation of HEXIM1 gene transcription and protein levels, resulting in re-sequestration of active P-TEFb (4, 33, 34).

**Fig 4:**
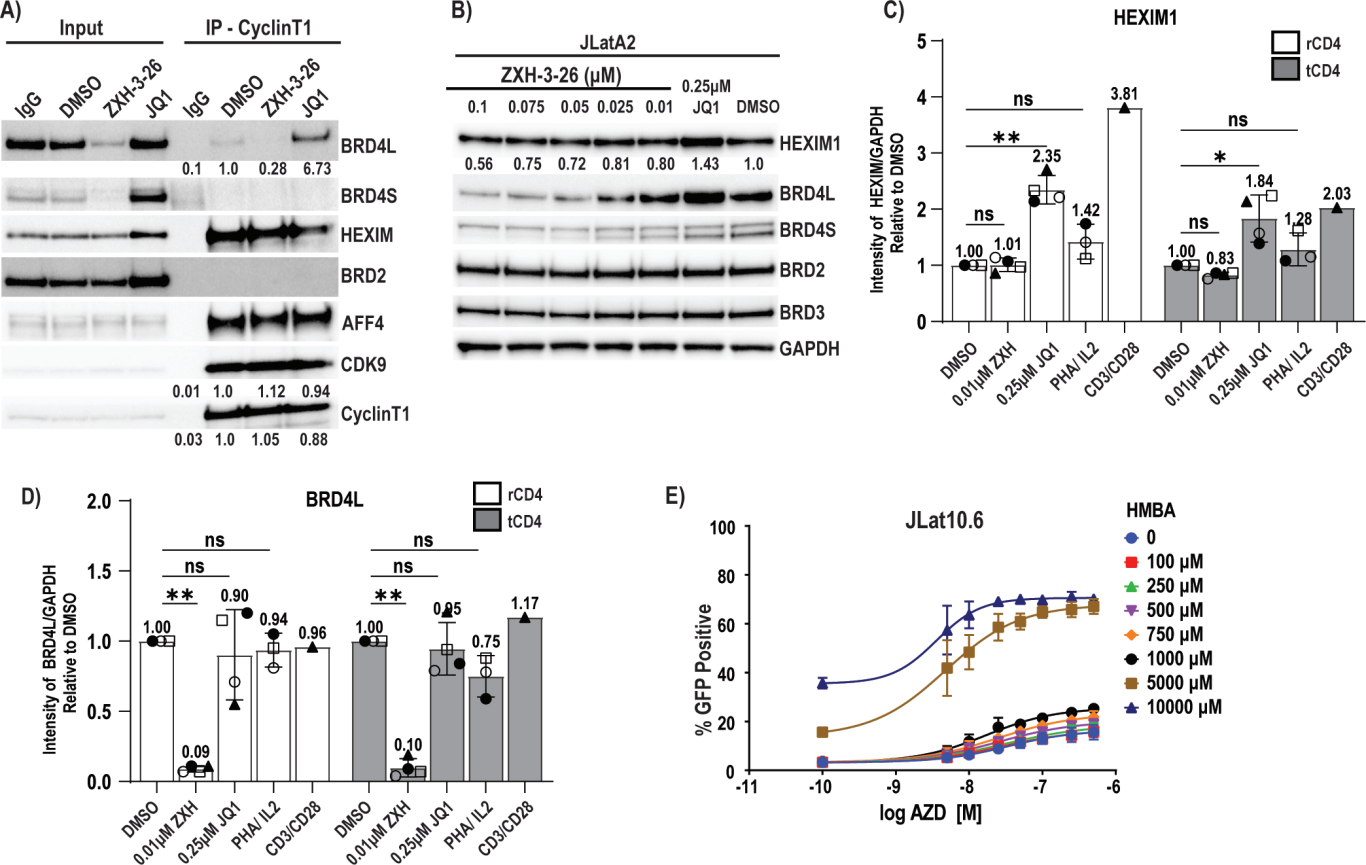
HEXIM1 is upregulated in response to BETi but not BET PROTACs. (**A**) Jurkat cells were treated with vehicle control (DMSO), 0.05 µM ZXH-3-26, or 0.5 µM JQ1 for 24 hours followed by a CyclinT1 or IgG immunoprecipitation and western blot for associated proteins. Relative protein levels are provided standardized to the DMSO IP control. HEXIM1 protein levels are upregulated in (**B**) JLatA2 cells in response to JQ1 but not in response to ZXH-3-26. (**C**) This observation is repeated in both resting and total primary CD4+ T-cells in cells from healthy donors (*n* = 4, SD). Relative protein levels were determined by standardizing each protein to the GAPDH loading control, followed by expression relative to the DMSO control. (**D**) Relative BRD4L(ong) levels in treated rCD4 and tCD4 (*n* = 4, SD). (**E**) Latency reversal in JLat10.6 cells with an 8-point dose titration of AZD5582 and hexamethylene bisacetamide (HMBA). Data represents three experiments of each 8-point titration (*n* = 3, SD). Independent replicates and extended dose curves are provided in Fig. S6. For (**C**) and (**D**), significance was assessed relative to DMSO control by Kruskal-Wallis with uncorrected Dunn’s multiple comparison test, (*) *P* < 0.05, (**) *P* < 0.01, (***) *P* < 0.001, (****) *P* < 0.0001.

As expected, CyclinT1 co-immunoprecipitated with BRD4, CDK9, HEXIM, and SEC member AFF4 but not BRD2, a non-interacting control (Fig. 4A). JQ1 treatment resulted in increased BRD4 association with CyclinT1 at both 0.5 µM (Fig. 4A) and 0.25 µM (Fig. S6A). These results demonstrate BETi do not impact BRD4/P-TEFb interaction, consistent with the BRD4 CTD/PID and not the bromodomains as the primary interaction domain with CyclinT1 but inconsistent with a mechanism in which BRD4 inhibition reduces competition for P-TEFb with Tat. Meanwhile, ZXH-3-26 treatment abrogated BRD4 interaction with CyclinT1 (Fig. 4A), demonstrating BET degraders would effectively reduce competition between BRD4 and Tat for P-TEFb if this were the primary mechanism of latency reversal.

Interestingly, immunoprecipitation input samples demonstrated JQ1-induced HEXIM expression in contrast to ZXH-3-26 (Fig. 4A). Over the optimized dose range in JLatA2 cells, ZXH-3-26 at concentrations selective for BRD4 degradation did not induce increased HEXIM protein levels as compared to 0.25 µM JQ1, which increased 1.43× over the DSMO control (Fig. 4B). We observed similar results with MZ1 (Fig. S6B) at 0.01–0.1 µM and for both degraders at a greater range of concentrations (Fig. S6C). Importantly, we confirmed HEXIM upregulation by BETi but not BET degraders in both total and transcriptionally quiescent resting CD4+ T-cells (Fig. 4C). We assayed HEXIM levels by western blot in four healthy donors (Fig. 4C and Fig. S6D and G) and observed a 2.35-fold average increase in resting CD4+ T-cells (*P* < 0.01) and a 1.84-fold average increase in total CD4+ T-cells (*P* < 0.05) in response to 0.25 µM JQ1 as compared to the vehicle control. In contrast, 0.01 µM ZXH-3-26 demonstrated no significant increase in HEXIM1 in either cell type (Fig. 4C). We confirmed BRD4 was significantly downregulated in ZXH-3-26 treated cells at levels comparable to prior results (Fig. 4D). While HEXIM upregulation is an indirect measure of P-TEFb release, these results strongly support the hypothesis that the primary difference between BET degraders and BETi is the disruption of P-TEFb from the 7SK RNP and implicates this as the primary mechanism in HIV latency reversal.

To further confirm BETi/IAPi synergy is primarily driven by P-TEFb disruption from 7SK, we hypothesized pairing AZD5582 with another disruptor of the P-TEFb pathway, hexamethylene bisacetamide (HMBA), would also result in synergistic latency reversal. Early studies of HMBA observed the upregulation of HEXIM mRNA as a primary result of treatment (35), an observation now understood to be a result of P-TEFb disruption (34, 36, 37). HMBA is also a known LRA requiring high mM concentrations for activity (38, 39). When combined with AZD5582, we observed weak synergy at concentrations ≤1 mM but observed strong synergy at 5 mM and 10 mM with suboptimal concentrations of AZD5582 in JLat10.6 cells (Fig. 4D and Fig. S6D), implicating P-TEFb release from 7SK as the primary driver of synergy with IAPi.

### Disruption of P-TEFb/7SK is dependent on the release of intact BRD4 from chromatin

**Fig 5:**
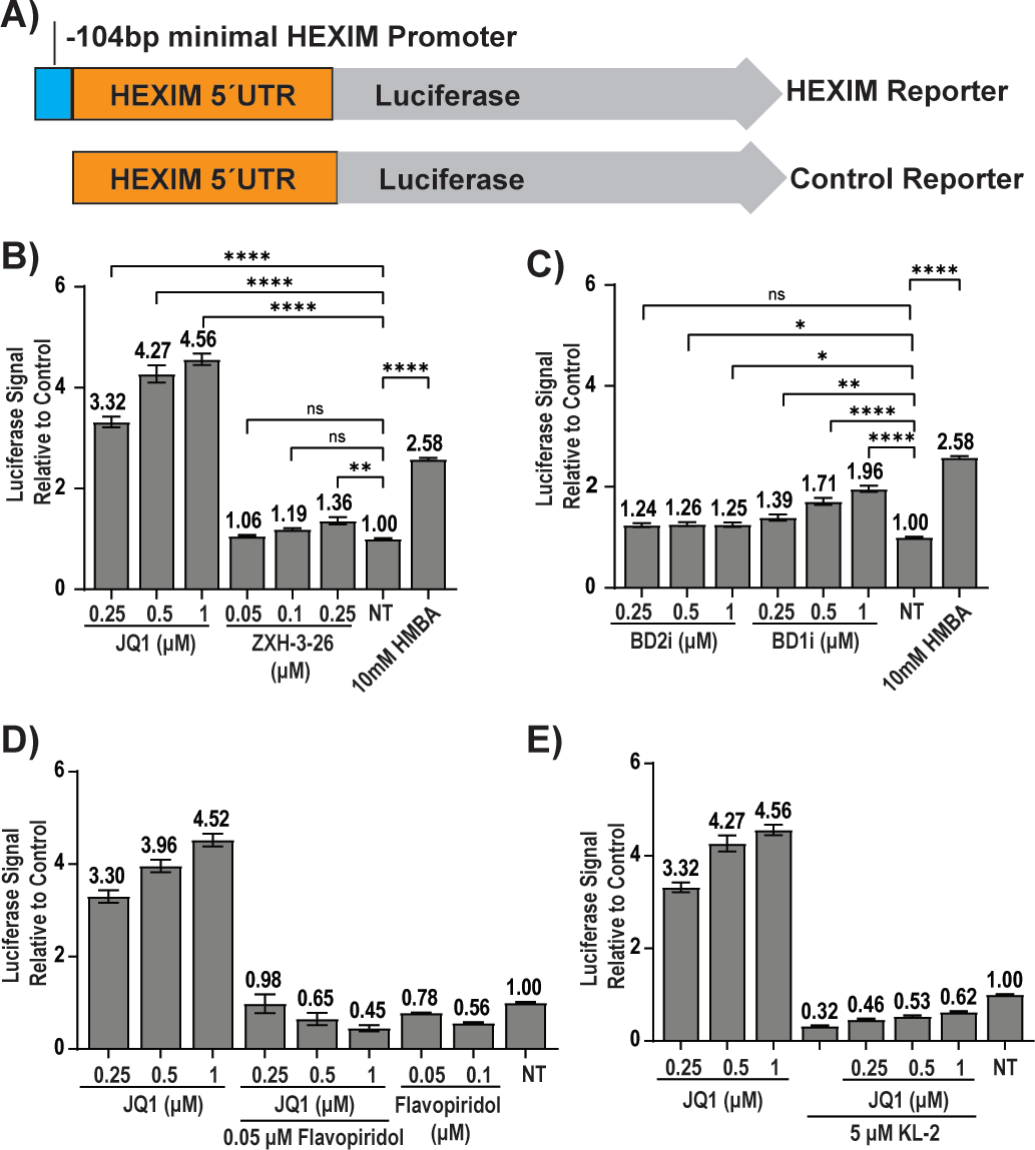
HEXIM1 induction occurs at the transcriptional level and requires the SEC. To generate 293T cell lines for high throughput assay of conditions that induce the HEXIM promoter, (**A**) the reported minimal HEXIM1 promoter and UTR or the UTR alone (control) were inserted before a luciferase reporter. Fold induction of luciferase is demonstrated for (**B**) JQ1 and ZXH-3-26 (three independent experiments, biological triplicates, *n* = 9, SEM) and for (**C**) BD1 or BD2 specific inhibitors (three independent experiments, biological triplicates, *n* = 9, SEM). Cells were treated at indicated concentrations for 24 hours and the luciferase signal standardized to no treatment (NT, DMSO control). Luciferase induction by JQ1 was blocked by concurrent treatment with (**D**) Flavopiridol (two independent experiments, biological triplicates, *n* = 6, SEM) and (**E**) KL-2 (three independent experiments, biological triplicates, *n* = 9, SEM). For (**B**) and (**C**), significance was assessed relative to NT (no treatment) control by Kruskal-Wallis with uncorrected Dunn’s multiple comparison test, (*) *P* < 0.05, (**) *P* < 0.01, (***) *P* < 0.001, (****) *P* < 0.0001.

The specific mechanism through which BETi disrupts P-TEFb from 7SK has not been described. Induction of the HEXIM transcript is reported to be a direct feedback loop dependent on P-TEFb and SEC components but independent of BRD4 (33, 40). To further probe the mechanism of JQ1-mediated P-TEFb release, we used HEXIM1 upregulation as a reporter. Lui et al. (33) had previously identified the minimal HEXIM promoter responsive to modulation of P-TEFb levels. We cloned the 104 bp of the HEXIM1 promoter identified by Liu et al. but also included the entire HEXIM1 untranslated region (UTR) upstream of luciferase and generated 293T/17 cell lines which stably expressed the minimal promoter construct (293T-HEXIMpr104) and a control reporter that only contained the UTR (293T-HEXIMpr0) (Fig. 5A). Preliminary characterization confirmed only the 293T-HEXIMpr104 line was responsive to JQ1 and HMBA while Flavopiridol, a CDK9 inhibitor, reduced basal luciferase expression (Fig. S7A and B). JQ1 (0.25–1 µM) significantly induced luciferase expression (3.32×–4.56× induction over control, *P* < 0.0001, Fig. 5B), demonstrating that HEXIM protein induction occurs secondary to transcriptional upregulation and is not a result of stabilization of the protein. We next examined the impact of BET degraders on HEXIM1 transcriptional induction. ZXH-3-26 at a BRD4-selective dose of 0.05 µM and 0.1 µM failed to induce a response (Fig. 5B). At a higher, non-BRD4 selective dose of 0.25 µM, ZXH-3-26 treatment resulted in upregulation (1.2–1.36× over control, *P* < 0.01) but remained weaker than HMBA (2.58× induction, *P* < 0.001) and JQ1 (Fig. 5B). Taken together, these data demonstrate that BETi but not BET degraders mediate P-TEFb disruption from 7SK RNP as measured by HEXIM1 upregulation.

Next, we evaluated whether displacement of BRD4 from chromatin is necessary for P-TEFb disruption and subsequent HEXIM upregulation. The BET family contains tandem bromodomains, referred to as BD1 and BD2. Recently published BD-selective inhibitors have demonstrated a key role for BD1 in chromatin binding (41, 42). Inhibition of BD1 disrupts BET proteins from chromatin while BD2-selective inhibition retains DNA binding of the family (41, 42). We used these inhibitors to determine the contribution of each bromodomain to HEXIM induction. BD2-selective GSK-046A upregulated HEXIM (1.25× over the control, *P* < 0.05) but showed no dose-dependent increase in luciferase expression (Fig. 5C). In contrast, BD1-selective GSK-779A (41, 42) resulted in a significant dose-dependent increase in luciferase expression (Fig. 5C, *P* < 0.0001 at 1 uM). These observations correlate with our prior results that BD1-selective inhibitors, but not BD2-selective, induce latency reactivation in an HIV latency model (16). These results indicate that the release of BRD4 from chromatin is necessary for P-TEFb disruption and activation of both HEXIM and HIV. We further assayed the ability of CDK9 inhibitor Flavopiridol and compound KL-2 (43), which inhibits the AFF4/P-TEFb/SEC interaction, to block HEXIM upregulation by JQ1. While both compounds exhibited toxicity (Fig. S7E and F), both blocked the induction of luciferase by JQ1 (Fig. 5D and E), confirming that P-TEFb and AFF4 have roles in HEXIM upregulation (33).

### BRD4 is required for the upregulation of HEXIM transcription

**Fig 6:**
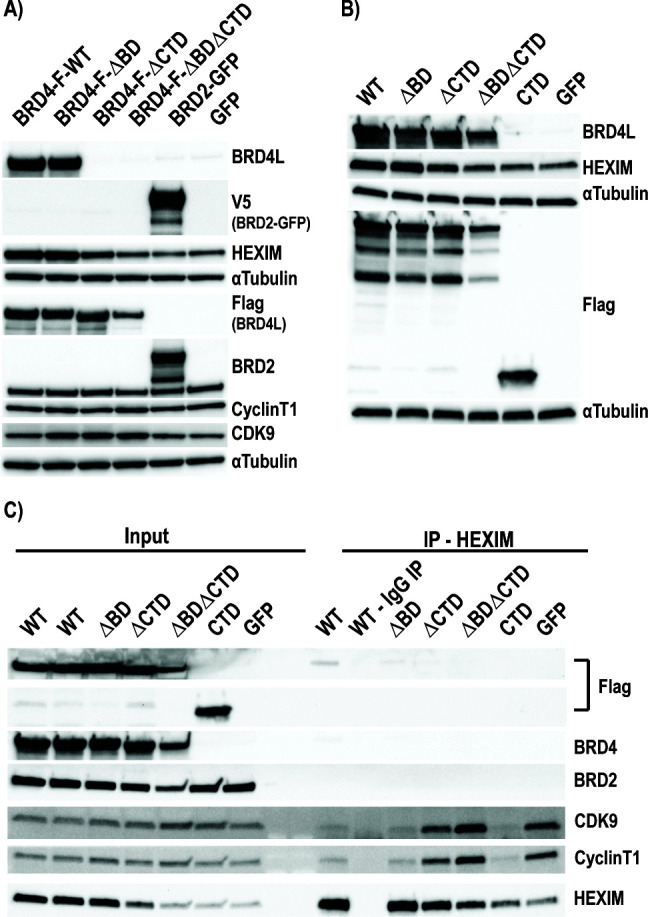
P-TEFb disruption from 7SK is dependent on the CTD but is independent of the BD domains. (**A**) Overexpression plasmids containing various full-length flag-tagged BRD4 constructs or (**B**) full-length and CTD-only constructs were transfected into 293T cells for 48 hours to determine the impact on HEXIM1 protein levels. (**C**) Overexpression constructs containing full-length WT, mutant, or the CTD domain alone were transfected into 293T cells for 48 hours and followed by a HEXIM1 or control IgG immunoprecipitation and western blot for associated proteins. Independent replicates for all blots are provided in Fig. S8.

These data implicate BRD4 release from chromatin as critical for P-TEFb disruption by BETi; however, degradation of BRD4 abrogates HEXIM1 upregulation and associated latency reversal activity. Collectively, this suggests that while bromodomain inhibition and subsequent chromatin displacement of BRD4 are important, other domains of BRD4 play a critical role in the actual disruption of P-TEFb from 7SK RNA, HEXIM upregulation, and latency reversal. In addition to the well-characterized CTD/PID (7, 44), the BD domains, specifically BD2, have also been implicated in binding acetylated CyclinT1 and necessary for latency reversal (45); however, the role of these domains in HEXIM upregulation is unknown. Therefore, we next determined the contribution of the BD domains and CTD to HEXIM protein induction by overexpressing wild-type BRD4 (BRD4-F-WT), a mutant with non-functional BD domains (BRD4-F-ΔBD), a mutant with the CTD/PID deleted (BRD4-F-ΔCTD), and a dual BD/CTD mutant (BRD4-F-ΔBDΔCTD) (Fig. 6A). A V5-tagged BRD2-GFP fusion and GFP overexpression constructs were transfected as controls (Fig. 6A). All mutant constructs expressed with only the BRD4-F-ΔBDΔCTD showing reduced protein levels when assayed by western blot (Fig. 6A). Of the expressed proteins, only BRD4-WT and BRD4-F-ΔBD were capable of inducing HEXIM expression (Fig. 6A and Fig. S8A), demonstrating the BD domains are not necessary for this activity. BRD4-F-ΔCTD showed upregulation; however, since this construct has intact BD domains, it is possible that it could displace endogenous WT BRD4 from chromatin, resulting in disruption of P-TEFb. Previously, overexpression of the CTD alone (PID aa1209-1362) was shown to disrupt P-TEFb from HEXIM and inhibit Tat-based elongation of HIV (7, 44, 45). Interestingly, we observed overexpression of the CTD/PID alone did not induce HEXIM protein expression (Fig. 6B and Fig. S8B).

We then performed HEXIM immunoprecipitations to determine which constructs could result in loss of P-TEFb/HEXIM association in the context of the 7SK RNP to determine how this correlated to HEXIM protein induction. BRD4-WT, BRD4-F-ΔBD, and the CTD/PID alone resulted in a loss of both CyclinT1 and CDK9 association with HEXIM (Fig. 6C and Fig. S8C), irrespective of induced HEXIM protein levels as observed in the input samples. These results confirm that CTD/PID is required and sufficient for disruption of P-TEFb from the 7SK RNP, but further find that HEXIM induction at the transcriptional level is also dependent on intact BRD4 protein and cannot be induced by overexpression of the CTD/PID alone. These data suggest additional protein-protein interactions are required to induce HEXIM upregulation and may also be required for HIV latency reversal.

### Latency reversal by BETi in Jurkats is dependent on CDK9 but not the SEC

**Fig 7:**
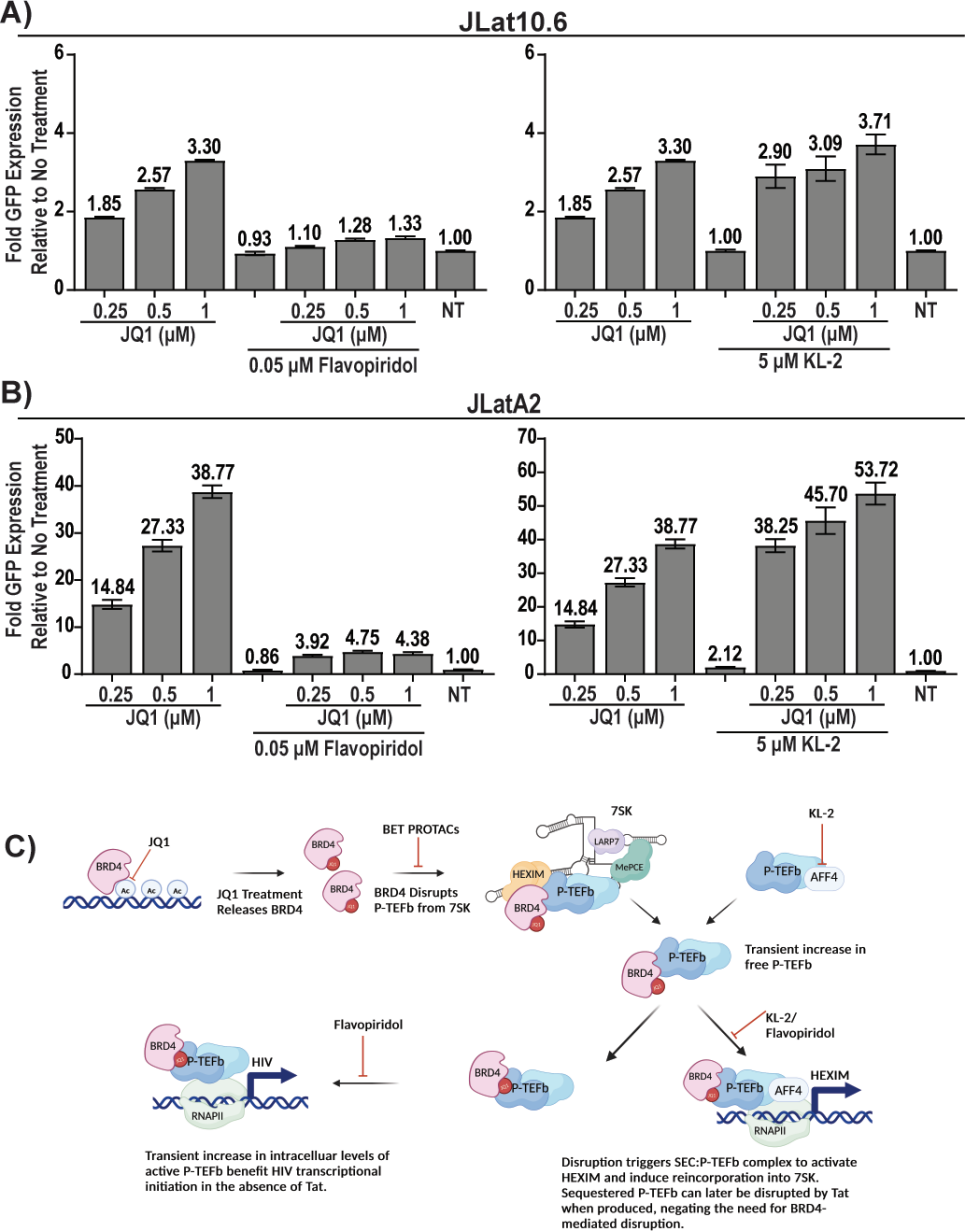
Latency reversal by JQ1 is blocked by Flavopiridol but not KL-2. In both (**A**) JLat10.6 and (**B**) JLatA2 cells, latency reversal as measured by fold GFP induction by JQ1 is blocked by CDK9 inhibitor but enhanced by SEC inhibitor KL-2 (two independent experiments, biological triplicates, *n* = 6, SEM). (**C**) Proposed mechanism of BETi-mediated latency reversal in the absence of Tat.

Finally, we moved back into both the JLatA2 and JLat10.6 latency models to determine if the same determinants of HEXIM induction correlated to latency reversal. As expected, Flavopiridol inhibited latency reversal induced by JQ1 in both cell lines (Fig. 7A and 7B). Unexpectedly, the SEC inhibitor KL-2 enhanced JQ1-mediated latency reversal (Fig. 7A and 7B). Thus, while HEXIM1 transcriptional induction is dependent on the SEC, these results demonstrate that JQ1-induced latency reversal of HIV is independent of the SEC and that disruption of other canonical P-TEFb partners beyond the 7SK RNA benefits viral activation in JLat models.

## DISCUSSION

Here, we demonstrate two bivalent small molecule degraders, ZXH-3-26 and MZ1, fail to induce latency reactivation in full-length JLat models and in cells from ART-suppressed donors despite highly selective and robust BRD4 degradation. These observations are in direct conflict with a model in which BRD4 at the HIV LTR acts to repress and compete against Tat for P-TEFb. Rather, we observed the primary difference between BRD4 degradation versus inhibition was the upregulation of HEXIM transcription and protein levels, a pathway known to be upregulated by the release of P-TEFb from the repressive 7SK complex (4, 33, 34). We further find evidence that disruption of other canonical P-TEFb complexes, specifically the SEC/P-TEFb complex by inhibitor KL-2, further enhances latency reversal by BETi. Consistent with our observations, Ciscernos et al. have recently demonstrated in greater mechanistic detail that KL-2 disrupts cellular SEC/P-TEFb complexes and synergizes with BETi and other LRAs to activate HIV from latency (46).

We propose an updated model, in which the release of BRD4 from chromatin by BD-domain inhibition allows BRD4 to interact via the CTD/PID with P-TEFb in 7SK, transiently increasing free P-TEFb levels (Fig. 7C). This disruption of P-TEFb equilibrium activates a CDK9 and SEC-dependent feedback loop that triggers upregulation of the repressive protein HEXIM to reincorporate and re-repress dysregulated P-TEFb. Concurrently, the disruption and increased P-TEFb also trigger HIV LTR activation that is CDK9 dependent, but, in the context of Jurkat-derived models containing Tat, is independent of the SEC (Fig. 7C). The increased BRD4/P-TEFb complex may result in sufficient HIV transcriptional activation to drive Tat protein expression, at which point eventual loss of the small molecule combined with HEXIM upregulation restores the 7SK/P-TEFb pool, allowing Tat to access P-TEFb and further drive HIV transcription. BRD4 is also reported to have intrinsic kinase activity and can phosphorylate the RNAPII CTD and CDK9 (47, 48), an activity that may further benefit HIV activation.

This mechanism reconciles observations from numerous studies in the HIV field related to BRD4 and latency reversal. We find that while the CTD/PID can indeed disrupt P-TEFb from 7SK, it cannot activate HEXIM transcription, suggesting targeting newly released P-TEFb back to chromatin and, by extension, HIV, is dependent on full-length BRD4 but not the bromodomains or histone binding. In support of this, a recent study observed an increased association of CDK9 with active transcriptional start sites and enhancers upon JQ1 treatment via chromatin immunoprecipitation (ChIP)-seq, even in the context of BRD4 loss from chromatin but not upon treatment with degrader dBET6 (27). The mechanisms of BRD4-mediated P-TEFb retargeting to chromatin and transcriptional activation in the context of bromodomain inhibition merit additional investigation to understand the BRD4/P-TEFb axis and HIV reactivation.

Furthermore, the arginine-rich RNA binding domain of Tat has been shown to interact with stem loop 1 of 7SK, allowing Tat to directly disrupt and recruit P-TEFb from 7SK (44, 49). However, Bisgrove et al. demonstrated Tat cannot displace P-TEFb from the BRD4/P-TEFb complex (7). We would postulate overexpression of BRD4 or the CTD/PID alone sequesters P-TEFb into a complex that Tat cannot disrupt. Prolonged overexpression of full-length BRD4 or the CTD/PID results in chronic depletion of P-TEFb from 7SK, regardless of HEXIM activation. We demonstrate overexpression of BRD4 after 48 hours results in strong depletion of endogenous P-TEFb from 7SK (Fig. 6). This could result in the observations that BRD4 and/or CTD/PID overexpression is refractory to Tat-mediated HIV activation (6, 7, 45).

Finally, we find that HEXIM protein expression can be induced in resting primary CD4+ T-cells by the BETi JQ1 despite prior observations of highly restricted CyclinT1 levels in resting cells (50–52). These results imply the presence of a 7SK-bound P-TEFb pool, likely limited, that can be disrupted with BET inhibitors. We acknowledge the limitations of this study, specifically the use of HEXIM as an indirect measure of P-TEFb release. We cannot rule out that an additional pathway, critical for HIV expression but unrelated to P-TEFb, responds differently to BET inhibition versus BRD4 degradation. However, the central role of P-TEFb in Tat function and HIV transcription makes this seem unlikely. Future studies to identify the specific mechanism and interaction partners involved in BRD4-mediated P-TEFb recruitment and elongation in the absence of histone binding will be informative. We propose that reimagining BRD4 as an activator rather than a repressor in the context of viral latency will reveal new mechanisms that can be exploited for HIV cure strategies.

## MATERIALS AND METHODS

### Cell lines

JLatA2 and JLat10.6 (26, 30) were obtained from the NIH AIDS Reagent Program. Cells were maintained in RPMI1640 (LifeTech) supplemented with 10% fetal bovine serum (FBS) (Millipore) and 100 U/mL Pen/Strep (LifeTech) at 37°C/5% CO_2_. HEK293T/17 cells were obtained from ATCC (CRL-11268) and maintained in DMEM (LifeTech) supplemented with 10% FBS (Millipore) and 100 U/mL Pen/Strep (LifeTech) at 37°C/5% CO_2_.

### CD4+ T-cell isolation

Standard isolation of peripheral blood mononuclear cells via Ficoll-Paque (GE Lifesciences) was performed on samples from anonymous, healthy blood donors (New York Blood Center). Total or resting CD4+ cells were obtained using either the EasySep Human CD4+ T-Cell Isolation or EasySep Human Resting CD4+ T-cell Isolation kits (Stemcell Technologies) per manufacturer protocol. To assess purity (3 of 4 donors), cells were stained with anti-CD4 (Clone RPA-T4), anti-CD3 (Clone UCHT1), anti-CD25 (Clone BC96), and anti-CD69 (Clone FN50) and run on the Attune NXT cytometer and analyzed using FlowJo. Representative gating scheme, FMOs, and CD25/CD69 levels of isolated cells are provided in Fig. S9.

### Latency reversal agents

Compounds were dissolved in DMSO unless indicated. For high-throughput flow and synergy assays, an HP D300e digital dispenser using T8+ or D8+ dispense heads was used to plate DMSO-soluble compounds. ZXH-3-26 (6713, CAS 2243076-67-5) was obtained from Tocris Bioscience. MZ1 (HY-107425, CAS: 1797406-69-9), *cis*MZ1 (HY-107425A, CAS: 1797406-72-4), and KL-2 (HY-123972, CAS: 900308-51-2) were purchased from MedChemExpress LLC. HMBA (aqueous) (H4663, CAS: 564468-51-5) was purchased from Millipore Sigma. Flavopiridol (L86-8275, CAS: 146426-40-6) was purchased from Selleckchem. (+)-JQ-1 was purchased from AstaTech Inc. (Catalog #: 41223, CAS: 1268524-70-4). (−)-JQ-1 was obtained from the SGC Oxford [(−)-JQ-1/SGCBD01, CAS: 1268524-71-5]. Recombinant human TNF-alpha (aqueous) (210-TA-020) was purchased from R&D Systems. AZD5582 (CT-A5582, CAS: 1258392-53-8) was purchased from ChemiTek. GSK-046A (BD2-specific) (41) and GSK-789A (BD1-specific) (42) were provided by GlaxoSmithKline. Purified PHA (aqueous) (R30852801) was purchased from ThermoFisher. Recombinant IL-2 (aqueous) (200-02) was purchased from Peprotech.

### Latency reversal/flow cytometry

Cells were plated in 96-well plates at 50,000/well and treated with degraders/inhibitors for 24 hours. For 16-point titrations, cells were treated at 0, 0.001, 0.005, 0.01, 0.025, 0.05, 0.1, 0.25, 0.5, 0.75, 1, 2.5, 5, 7.5, 10, and 20 µM. For all other experiments, cells were treated at indicated concentrations. N indicates the total number of biological replicates performed over three independent experiments. Cells were stained with LIVE/DEAD Fixable Aqua Dead Cell Stain (ThermoFisher) for 30 minutes, followed by DPBS wash and fixation in 1.5% paraformaldehyde/DPBS. Cells were assayed using the iQue Screener Plus (Intellicyt) and GFP expression with dead-cell exclusion was calculated using the ForeCyt analysis software (Intellicyt). Viability calculations represent total non-aqua staining cells as a percentage of the total cell gate.

### Western blots

Blots were performed as previously described (53) with minor modification: proteins were separated by 4%–20% TGX tris-glycine or 3%–8% Criterion XT tris-acetate gels with tris/glycine/sds or tricine buffers, respectively (BioRad), depending on size, and membranes were developed using Supersignal West Atto or West Pico (ThermoFisher). Antibodies: BRD4L (Bethyl A700-005), BRD4L/S (Abcam ab128874), BRD2 (Abcam ab139690), BRD3 (ab50818), HEXIM1 (CST 12604), CDK9 (CST 2316S), CyclinT1 (CST 81,464S), GAPDH (Abcam ab83956), aTubulin (Abcam ab7291), LaminB1 (Abcam ab133741), TBP (CST 44,059S), Flag (Sigma F1804), and V5 (Abcam ab27671). Volumetric analysis was performed using BioRad ImageLab software. For all westerns, relative intensity was standardized to the matched loading control, and values are shown relative to the vehicle control.

### Synergy

An 8-point cross titration of AZD5582 (0, 0.005, 0.01, 0.025, 0.05, 0.1, 0.25, and 0.5 µM) and JQ1 (0, 0.005, 0.01, 0.025, 0.05, 0.1, 0.25, and 0.5 µM), ZXH-3-26 (0, 0.005, 0.01, 0.025, 0.05, 0.1, 0.25, and 0.5 µM), or HMBA (0, 0.1, 0.25, 0.5, 0.75, 1, 5, and 10 mM) was performed in triplicate. Compounds were incubated for 24 hours and latency reversal was assayed via flow cytometry as described above. We calculated synergy based on the Bliss Independence model (54) like previously reported (55, 56) but used the maximal observed GFP induction in response to TNFα for each cell line to standardize the calculated synergy value in order to allow comparison between lines. For this work, F_a_(AZD5582, JQ1, ZXH3-26, or HMBA) = (Fraction GFP single agent – Fraction GFP DMSO)/TNFα max stimulation (JLatA2 or JLat10.6) and Fa_xy,O_ = (Fraction GFP combo – Fraction GFP DMSO)/TNFα-induced GFP max (JLatA2 or JLat10.6). Graphs displaying percent GFP positive in response to combinations are not normalized and represent raw GFP values.

### Luciferase reporter plasmids

We started with a previously generated pCDNA3-Luciferase construct generated in-house (A.-M. W. Turner, unpublished data). Briefly, pCDNA3-eGFP (a gift from Doug Golenbock, Addgene plasmid #13031; https://www.addgene.org/13031/; RRID: Addgene 13031) was digested with EcoRI and XbaI, and a PCR amplified luciferase with matching restriction sites was inserted in the place of GFP, generating pCDNA3-Luci. To generate the HEXIM reporter plasmids, pCDNA3-Luci was digested with MluI/BamH1 to remove the CMV promoter. To generate pDNA3-HEXpr104-Luci, the minimal 104 bp HEXIM1 promoter as reported in reference 33 was amplified from HEK293 cells along with the 716 bp HEXIM1 UTR with matching restriction sites and inserted via traditional restriction cloning. To generate the control pCDNA3-HEXpr0-Luci, only the HEXIM1 UTR was amplified and cloned. Resulting constructs were then transferred to an available lentiviral vector, pLKO.1-puro (a gift from Bob Weinberg, Addgene plasmid #8453; https://www.addgene.org/8453/; RRID: Addgene 8453) to generate stable lines. The U6 promoter was removed, and MluI/XbaI sites were added by deletion PCR. The HEXpr104-Luci or HEXpr0-Luci inserts digested from pCDNA3 were transferred via restriction digest cloning, generating pLKO.1-puro-HEXpr104-Luci and pLKO.1-puro-HEXpr0-Luci. At all stages, plasmids were confirmed via Sanger sequencing (Genewiz/Azenta Life Sciences).

### Luciferase reporter lines

Lentiviral particles were generated by transfecting 293T/17 either pLKO.1-puro-HEXpr104-Luci or pLKO.1-puro-HEXpr0-Luci, psPAX2 (a gift from Didier Trono, Addgene plasmid #12260; https://www.addgene.org/12260/; RRID:Addgene_12260), and pMD2.G (a gift from Didier Trono, Addgene plasmid #12259; https://www.addgene.org/12259/; RRID:Addgene_12259) using FugeneHD (Promega) per manufacturers protocol. Lentiviral particles were collected and filtered via a 0.45 um filter prior to transduction into fresh 293T/17 cells. Stable lines were selected using 1.5 ug/mL puromycin (Selleckchem) and maintained after selection under 0.5 ug/mL puromycin.

### Luciferase assays

Reporter lines were plated in clear bottom, white well 96-well plates and treated with compounds at indicated concentrations for 24 hours. To assess cellular viability, 10 uL of the resazurin-based PrestoBlue reagent (ThermoFisher) was added to each well, incubated at 37°C for 30 minutes, and fluorescence read using the SpectraMax M3 (Molecular Devices) using 555ex/585em with a 570 nm cutoff. Immediately following the plate read, PrestoBlue containing media was removed and 100 uL DMEM was added to all wells. Cells were immediately lysed in Steady-Glo (Promega) per manufacturer protocol, incubated for 15 minutes, and luminescence read on the SpectraMax M3.

### BRD4 overexpression plasmids

pCDNA5-Flag-BRD4-WT (a gift from Kornelia Polyak, Addgene plasmid #90331; https://www.addgene.org/90331/; RRID: Addgene 90331), pCDNA5-Flag-BRD4-BD (a gift from Kornelia Polyak, Addgene plasmid #90005; https://www.addgene.org/90005/; RRID: Addgene 90005), and BRD2-GFP (a gift from Kyle Miller, Addgene plasmid #65376; https://www.addgene.org/65376/; RRID: Addgene 65376) were purchased from Addgene. pcDNA5-Flag-BRD4-ΔCTD and pcDNA5-Flag-BRD4-BDΔCTD were generated via deletion PCR which removed amino acids 1330-1362. The pcDNA5-Flag-BRD4-CTD construct was generated via deletion PCR which removed amino acids 2-1208, leaving the 1209-1362 PID fragment previously described in (7, 44, 45). All BRD4 plasmids retained the N-terminal flag tag and were confirmed by Sanger sequencing (Genewiz/Azenta Life Sciences). Overexpression constructs were transfected into 293T cells for 48 hours using Fugene HD (Promega) per manufacturer’s instructions. For expression analysis, cell pellets were lysed in a modified RIPA as previously described (53) and subjected to western analysis.

### Immunoprecipitation experiments

For CyclinT1 IPs, Jurkat cells were treated with indicated concentrations of ZXH-3-26, JQ1, or DMSO vehicle control for 24 hours. For HEXIM IPs, 293T cells were transfected with overexpression constructs for 48 hours using Fugene HD per manufacturer’s instructions. Cell pellets were lysed in an NP-40 lysis buffer (25 mM Tris pH 7.5, 150 nM NaCl, 1% NP-40, 1× complete protease inhibitors (Millipore-Sigma), and 1× Halt Phosphatase Inhibitor cocktail (ThermoFisher) for 30 minutes on ice. Supernatant was cleared by centrifugation and protein concentration was assayed by detergent-compatible Bradford (Pierce/ThermoFisher). A volume of 5 uL anti-HEXIM (CST 1260) or 10 uL anti-CyclinT1 (CST 81,464S) was incubated with 500 ug total lysate for 1 hour at 4°C, followed by the addition of 1 mg of washed Dynabeads Protein G (ThermoFisher/Life Technologies) for an additional hour at 4°C. Beads were washed 5× in NP-40 lysis buffer and bound complexes were eluted at 95°C for 10 minutes using 50 uL of western loading buffer (1× Nupage LDS buffer + 1× Nupage Reducing Agent) (ThermoFisher). Eluted IP material and input samples were subject to western analysis. Volumetric analysis was performed using BioRad ImageLab software. Relative intensity was standardized to the vehicle control.

### HIV gag RNA induction in primary cells

Assays were performed as previously described in reference 16. Four to five replicates of 1–2E6 cells per donor per condition were assayed. Each point represents the average of these replicates per unique donor standardized to TBP except for PMA/Ionomycin. PMA/Ionomycin induces expression of all control genes, therefore induction is shown relative to DMSO and not standardized.

### Statistical analysis

All statistical analysis was performed using GraphPad Prism 10. Significance was assessed by Kruskal-Wallis with uncorrected Dunn’s multiple comparison tests whereby (*) *P* < 0.05, (**) *P* < 0.01, (***) *P* < 0.001, and (****) *P* < 0.0001.

## Data Availability

In accordance with the NIH DMS policy and the JVI data sharing policy, all raw data linked to this article can be located at https://dataverse.unc.edu/dataverse/turnerlab (https://doi.org/10.15139/S3/S1RBW5 and https://doi.org/10.15139/S3/4ESRC1).

## SUPPLEMENTAL MATERIAL

Supplemental material jvi.01777-24-s0001.docx Figures S1 to S9; Table S1.

ASM does not own the copyrights to Supplemental Material that may be linked to, or accessed through, an article. The authors have granted ASM a non-exclusive, world-wide license to publish the Supplemental Material files. Please contact the corresponding author directly for reuse.
