# Supplementary material for: BET degraders reveal BRD4 disruption of 7SK and P-TEFb is critical for effective reactivation of latent HIV in CD4+ T-cells
Source: J Virol. 2025 Mar 11;99(4):e01777-24. doi: 10.1128/jvi.01777-24 (PMC11998493; doi:10.1128/jvi.01777-24)
Supplement: Supplemental material — Figures S1 to S9; Table S1. [file jvi.01777-24-s0001.docx]

**Supplementary Figures**

**Supplementary Figure 1**

**
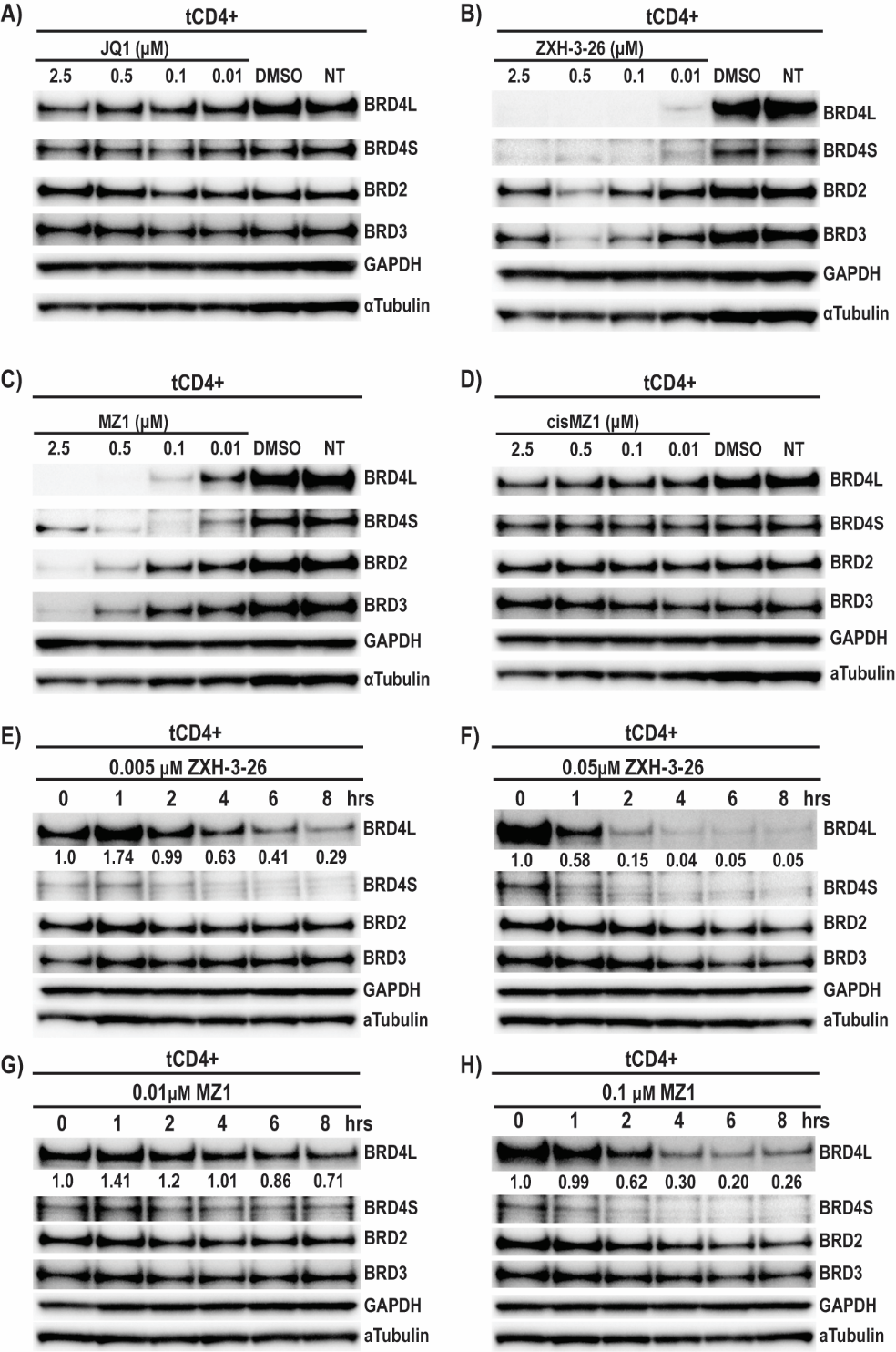
**

Figure S1 – *BET protein degradation in primary CD4+ T-cells* – Healthy primary human CD4+ T-cells (n=1 donor) were treated for 24 hrs at 0.01 µM, 0.1 µM, 0.5 µM, and 2.5 µM with **(A)** JQ1, **(B)** ZXH-3-26, **(C)** MZ1, and **(D)** control cisMZ1. BRD4L, BRD4S, BRD2, and BRD3 levels were assayed by western. GAPDH and αTubulin are provided as loading controls. To examine the timecourse of degradation, healthy primary human CD4+ T-cells (n=1 donor) were treated with **(E)** 0.005 µM ZXH-3-26, **(F)** 0.05 µM ZXH-3-26, **(G)** 0.01 µM MZ1, or **(H)** 0.1 µM MZ1. Cells were collected at 0 (immediately post dosing), 1, 2, 4 6, and 8 hours to assay BET protein levels. BRD4L levels are provided standardized to GAPDH relative to timepoint 0.

**Supplementary Figure 2**


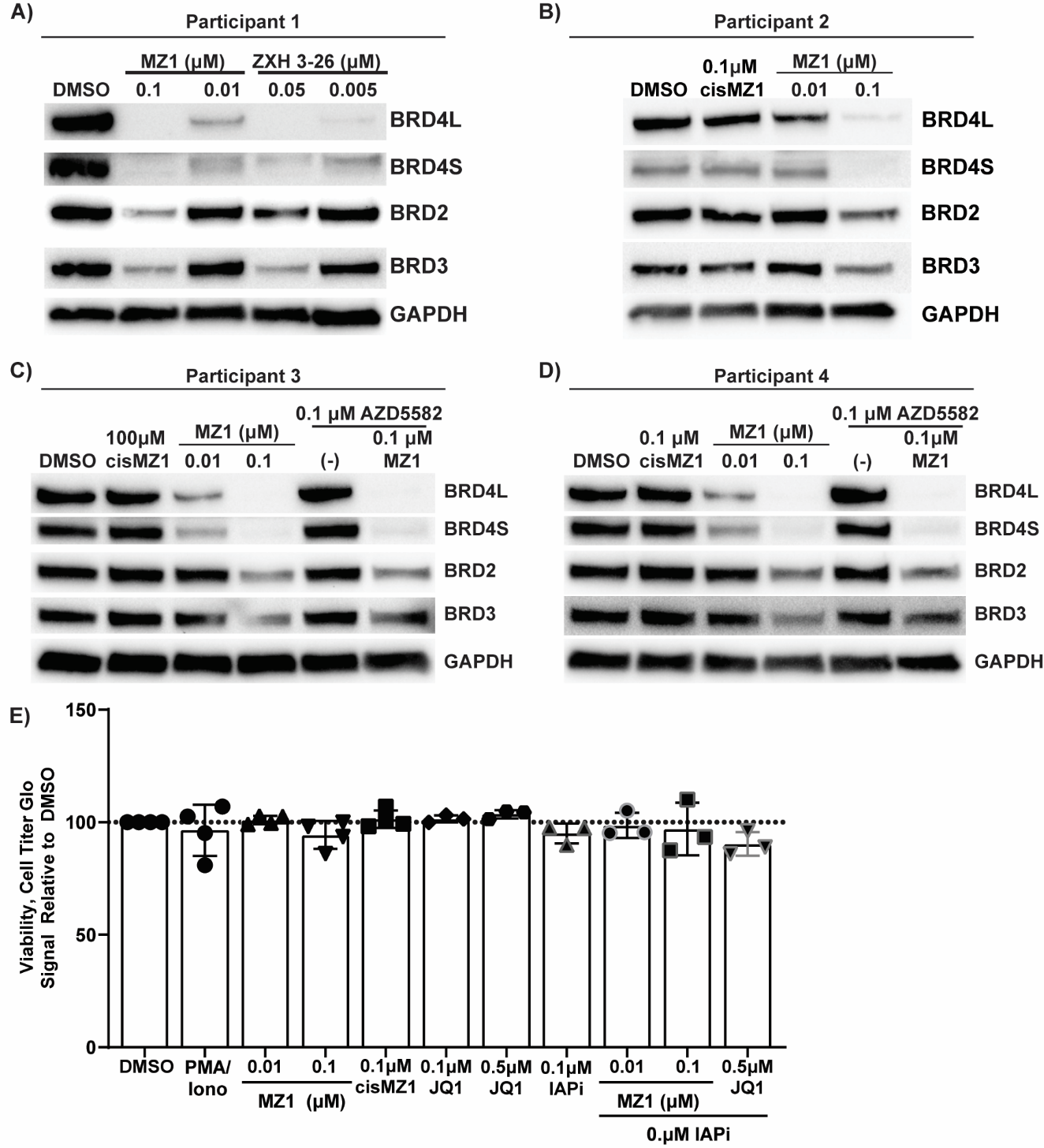


Figure S2 – *BET protein degradation by MZ1 in ART-suppressed donors* – 3 to 4E6 total CD4+ T-cells from ART-suppressed donors (n=4) were treated with MZ1 at 0.01 µM, 0.1 µM and vehicle control (DMSO) concurrent with cells treated for assessment of vRNA (Figure 1). Depending on availability of donor cells, 0.1 µM *cis*MZ1, 0.1 µM AZD5582, and AZD/MZ1 were also assayed. Degradation of BRD4L/S, BRD2, and BRD3 was assessed by western blot to confirm effective on-target degradation for donors 1-4 **(A-D)**. **(E)** Treated cells were assayed by CellTiter-Glo® per manufacturer’s instructions to assess cytotoxicity after MZ1 treatment (n=3 or 4, SD).

**Supplementary Figure 3**


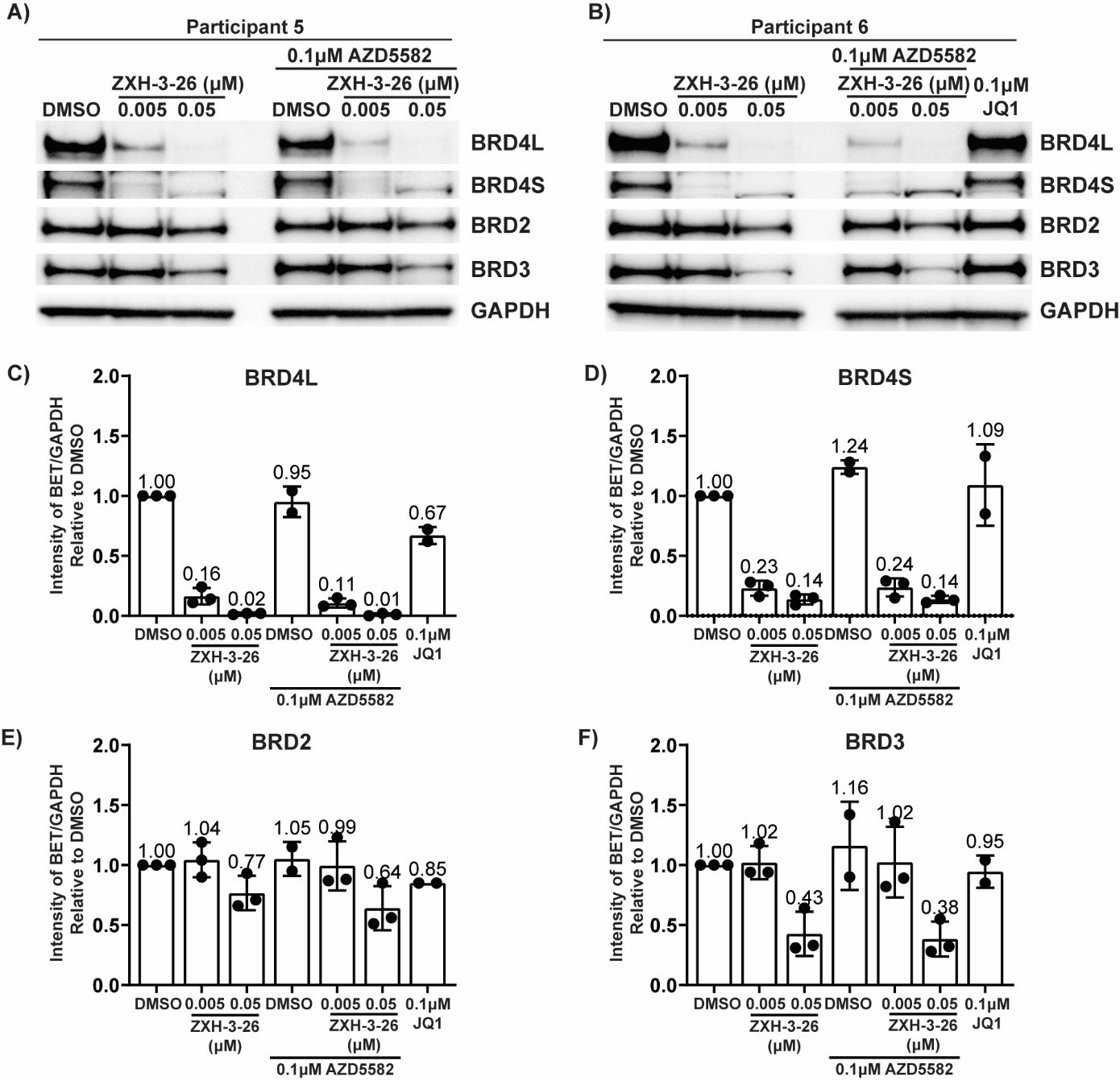


Figure S3 – *BET protein degradation by ZXH-3-26 in ART-suppressed donors* – 3 to 4E6 total CD4+ T-cells from ART-suppressed donors (n=3, SD) were treated with ZXH-3-26 at 0.005 µM, 0.05 µM and vehicle control (DMSO) concurrent with cells treated for assessment of vRNA. Depending on availability of donor cells, 0.1 µM AZD5582, and AZD/ZXH combinations, and JQ1 were also assayed. Degradation of BRD4L/S, BRD2, and BRD3 was assessed by western blot concurrent with cells treated for assessment of vRNA previously published in Falcinelli et al, 2022 (16). Western blots for **(A)** participants 5 and **(B)** 6 are provided. The western blot for participant 7 has been previously published Falcinelli et al, 2022 (16). Quantitation of **(C)** BRD4L, **(D)** BRD4S, **(E)** BRD2, and **(F)** BRD3 protein levels in response to ZXH-3-26 is provided for all three participants. ZXH-3-26 data from participant 1 in Supplementary Figure 2A was not included in this quantitation or previously published vRNA data.

**Supplementary Figure 4**


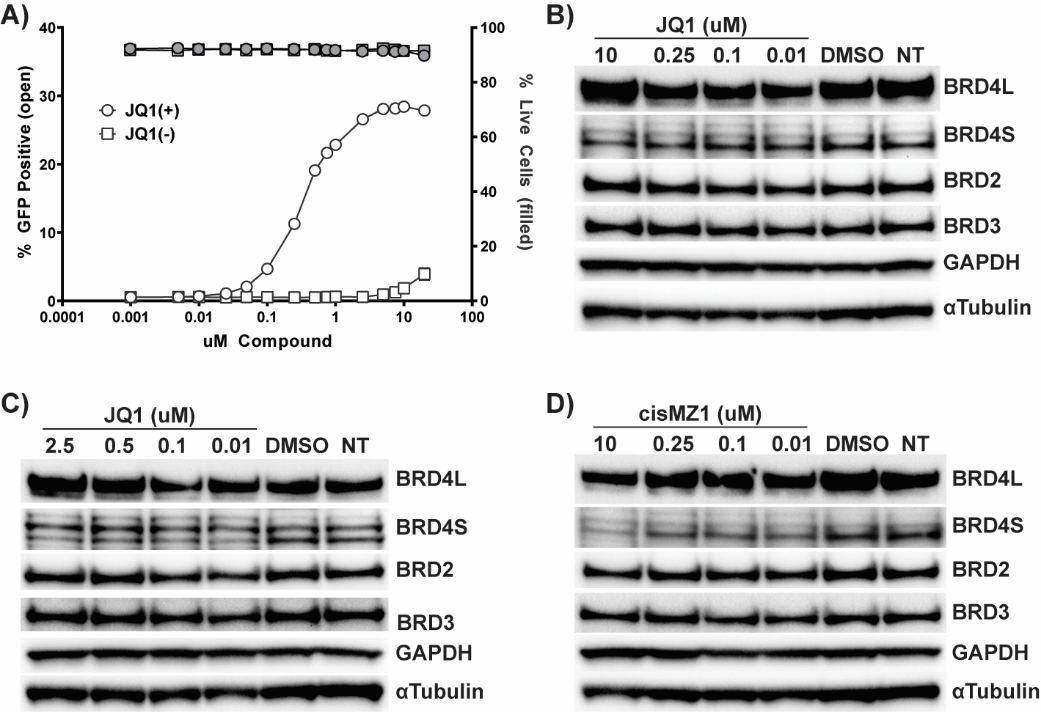


Figure S4 – *Latency reversal and targeted degradation of BET degraders in JLatA2 cells.* **(A)** Latency reversal by JQ1(+) versus inactive control JQ1(-) after treatment of JLatA2 cells with a 16-point dose titration for 24 hrs. Each titration was performed 2 independent times with biological triplicates for each experiment (n=6, SEM) with GFP (open symbols) assessed by flow cytometry as a measure of latency reactivation and viability by live/dead stain (gray symbols). Error bars not extending past the symbol are not visible. Protein levels in JLatA2 cells after treatment for 24 hrs with various concentrations of JQ1 **(B-C)** and cisMZ1 **(D)**. Data representative of two independent experiments.

**Supplementary Figure 5**


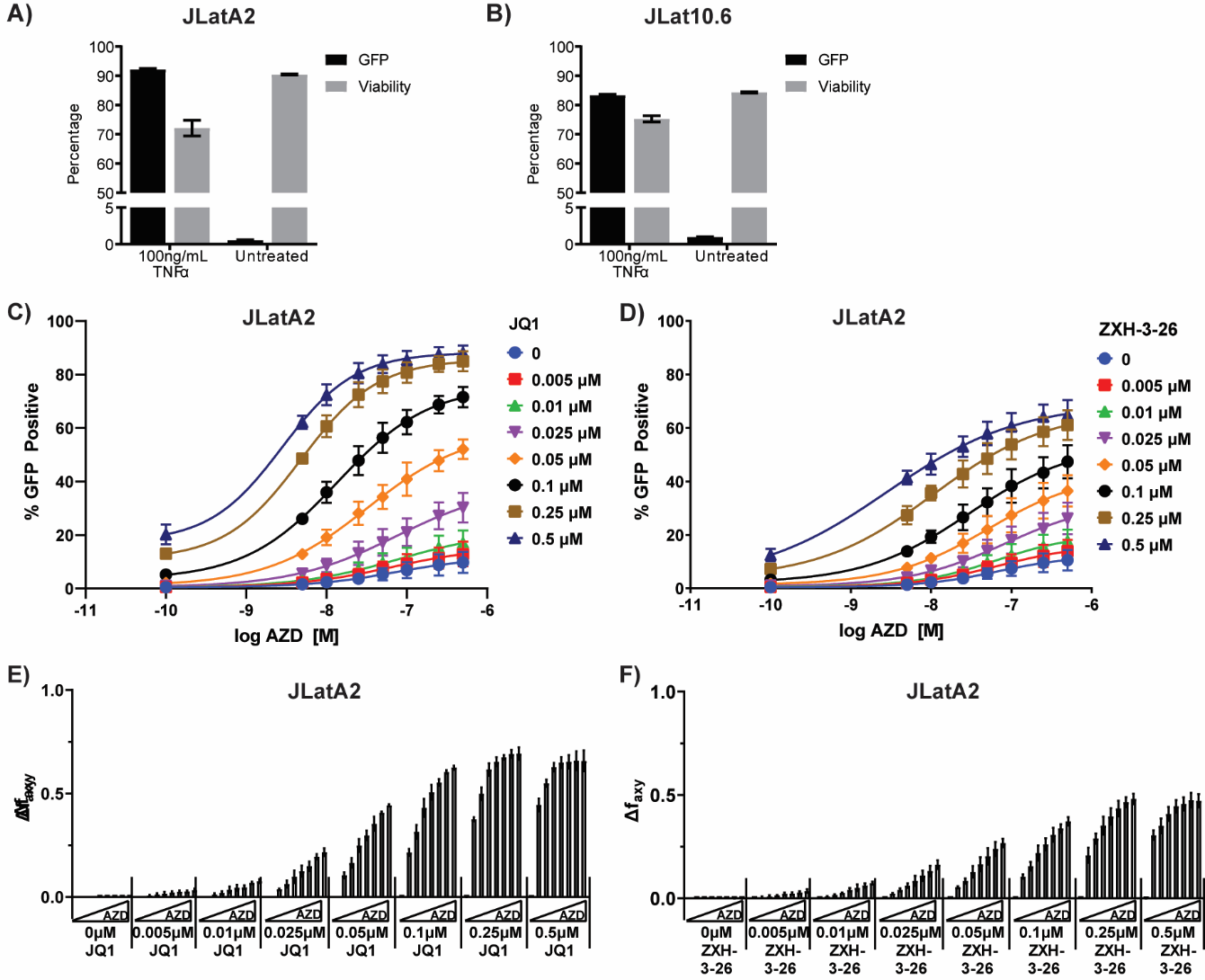


Figure S5 – *Supporting data for latency reversal and synergy in JLatA2 cells.* Comparison of GFP induction by 100 ng/mL TNFα in **(A)** JLatA2 (n=3, SD) and **(B)** JLat10.6 cells (n=3, SD). **(C)** 8-point dose titration of AZD5582 and JQ1 in JLatA2 cells. **(D)** 8-point dose titration of AZD5582 and ZXH-3-26 in JLatA2 cells. Data represents 3 independent experiments of each 8-point titration (n=3, SD). Bliss synergy calculations of **(E)** AZD5582/JQ1 and **(F)** AZD5582/ZXH-3-26 in JLatA2 cells from data in C/D (n=3, SD).

**Supplementary Figure 6**


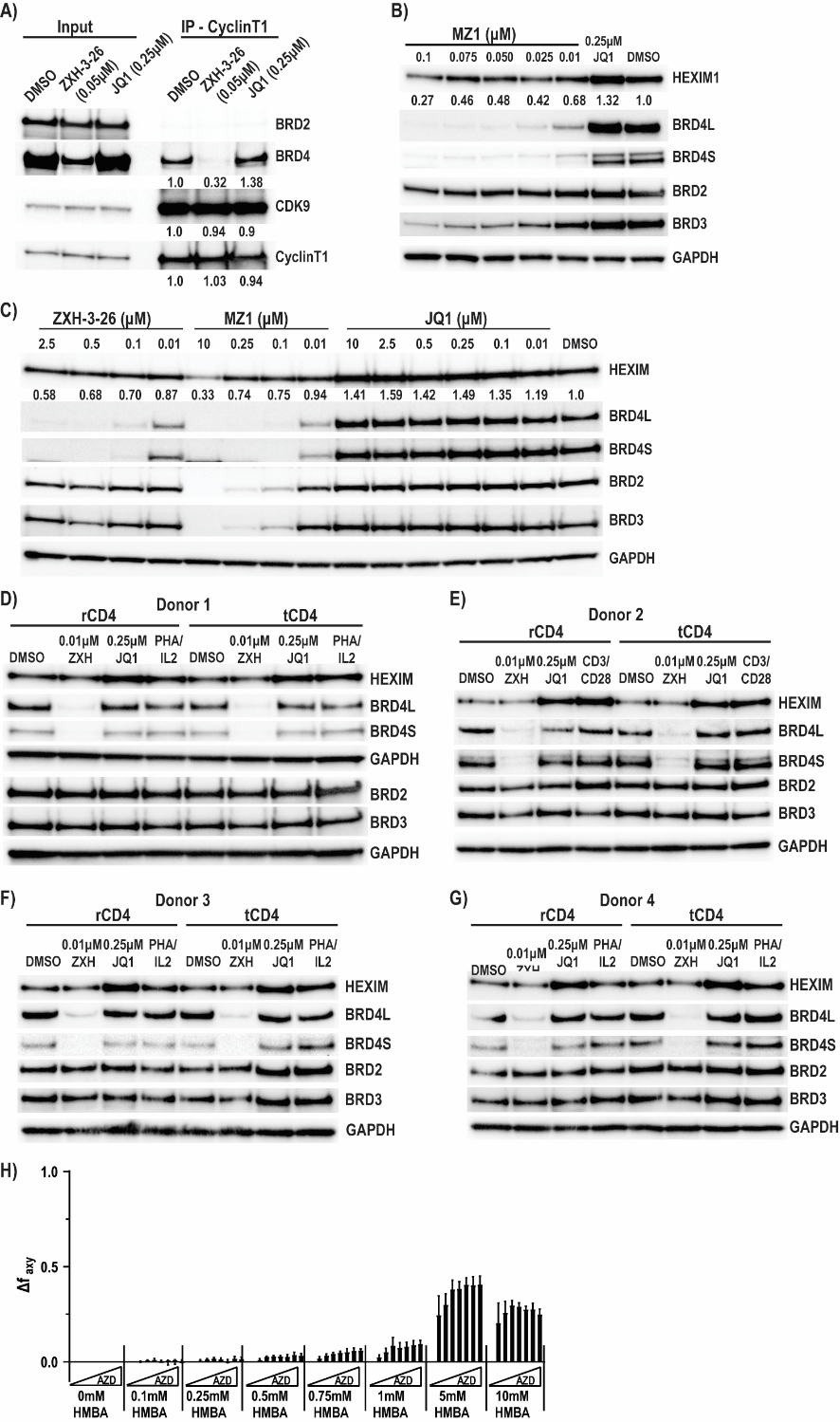


Figure S6 – *Impact of BETi or PROTACs on P-TEFb association with BRD4 and HEXIM upregulation –* **(A)** Jurkat cells were treated with vehicle control (DMSO), 0.05 µM ZXH-3-26, or 0.25 µM JQ1 for 24hrs followed by a CyclinT1 immunoprecipitation and western blot for associated proteins. Relative protein levels are provided standardized to the DMSO IP control. HEXIM1 protein levels are not induced in **(B)** Jurkat-derived cells in response to MZ1. This observation is repeated in **(C)** extended dose curves of BET PROTACS ZXH-3-26 and MZ1 as compared to BETi JQ1. **(D-G)** Western blots from 4 independent donors demonstrate HEXIM is upregulated in response to JQ1 treatment but not ZXH-3-26 in both resting and total CD4+ T-cells. **(H)** Bliss synergy is observed between AZD5582 and HMBA in JLat10.6 cells. Calculations from data provided in Figure 4E (n=3, SD).

**Supplementary Figure 7**


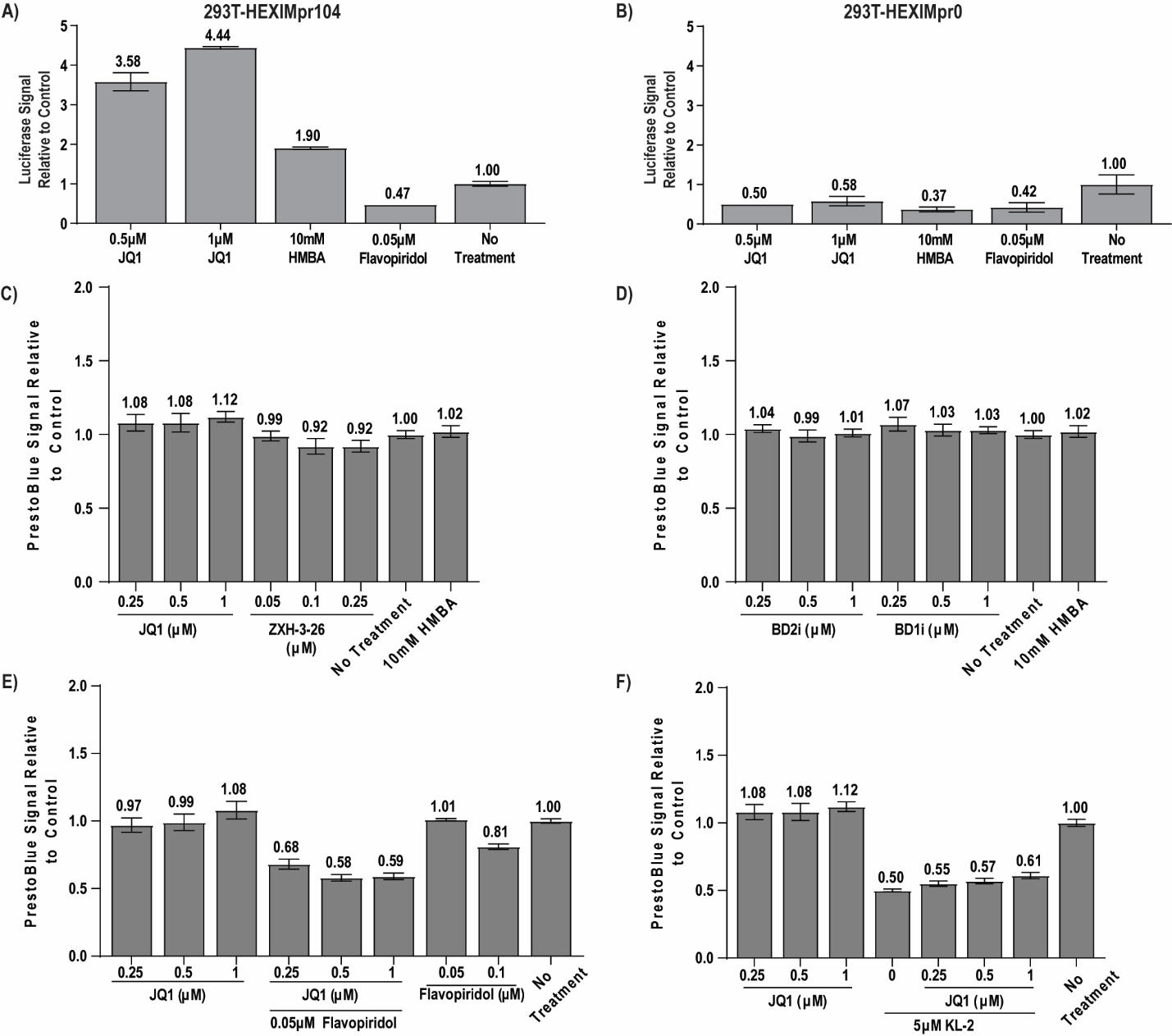


Figure S7 – *HEXIM1 Promoter Luciferase Assay –* **(A)** The minimal 104bp HEXIM promoter is sufficient and responsive to known disruptors of P-TEFb JQ1 and HMBA while **(B)** the control reporter containing only the HEXIM1 UTR fails to induce luciferase expression (n=3, SD). Viability of treated 293-HEXIMpr-Luc lines reported in Figure 7 measured by prestoblue **(C-F)**. Replicates and error are noted in Figure 7.

**Supplementary Figure 8**


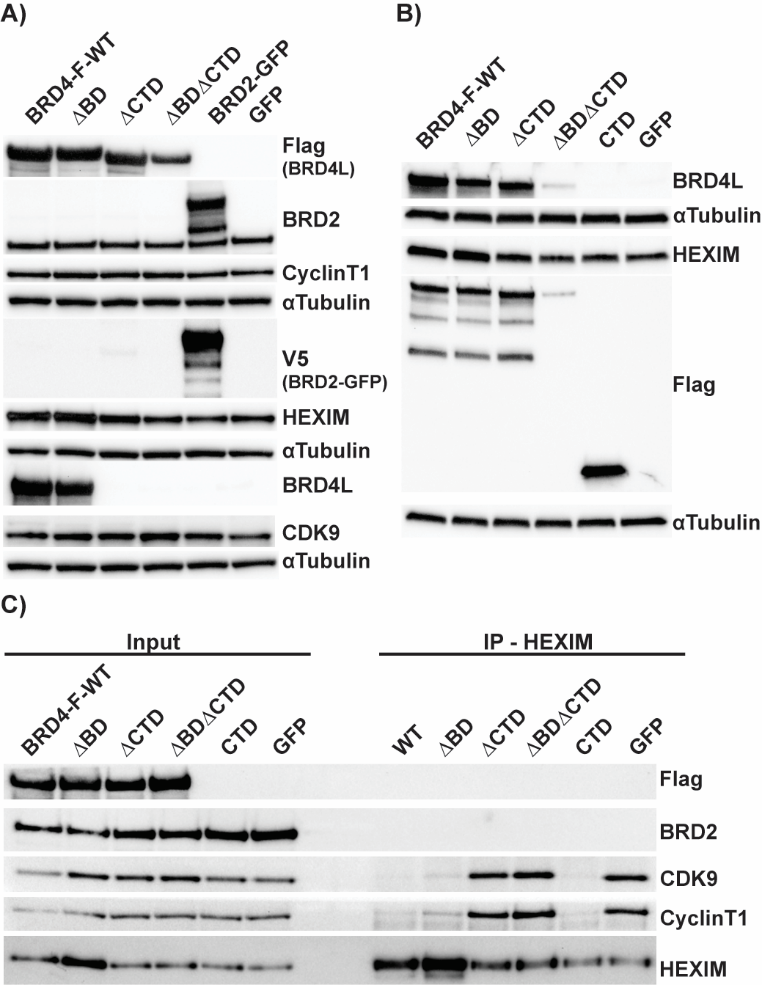


Figure S8 – *P-TEFb disruption from 7SK is dependent on the CTD but is independent of the BD domains* – Replicate experiments of **(A)** Overexpression plasmids containing various full-length flag-tagged BRD4 constructs or **(B)** full length and CTD-only constructs transfected into 293T cells for 48hrs to determine impact on HEXIM1 protein levels. **(C)** Overexpression constructs containing full length WT, mutant or the CTD domain alone were transfected into 293T cells for 48hrs and followed by HEXIM1 immunoprecipitation and western blot for associated proteins.

**Supplementary Figure 9**


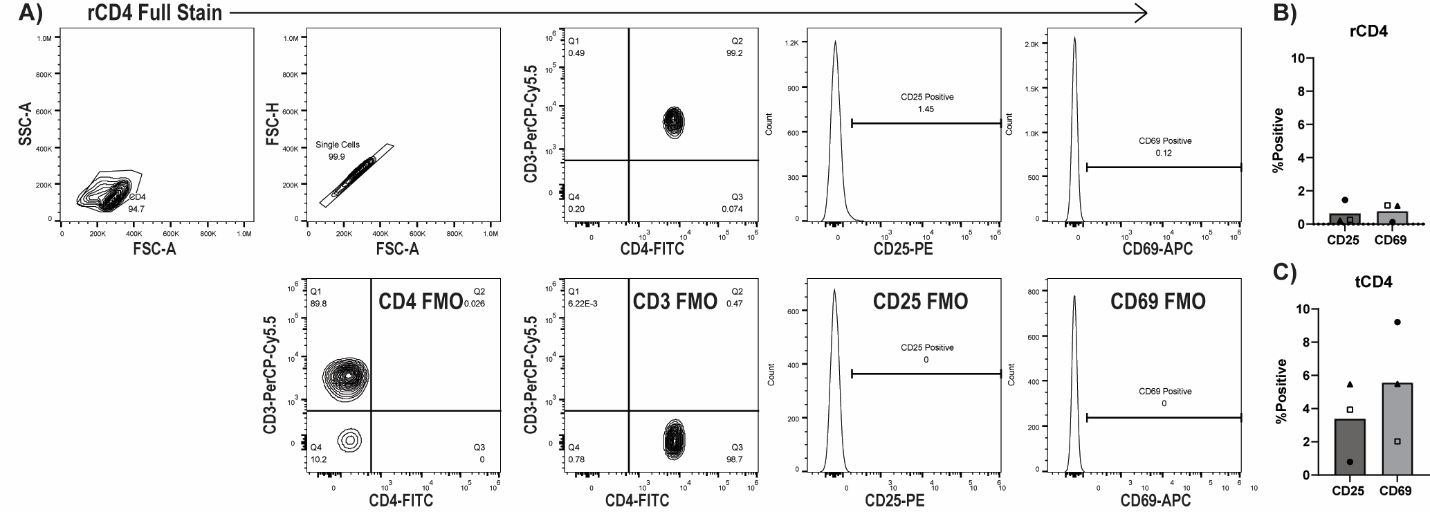


Figure S9 – **(A)** Flow gating scheme to determine purity of isolation of resting and total CD4+ T-cells isolated from healthy donors. CD25 and CD69 levels in **(B)** resting and (C) total CD4 cells used to examine HEXIM upregulation in response to JQ1 in n=3 of 4 donors. One donor was not assessed.

**Table S1**


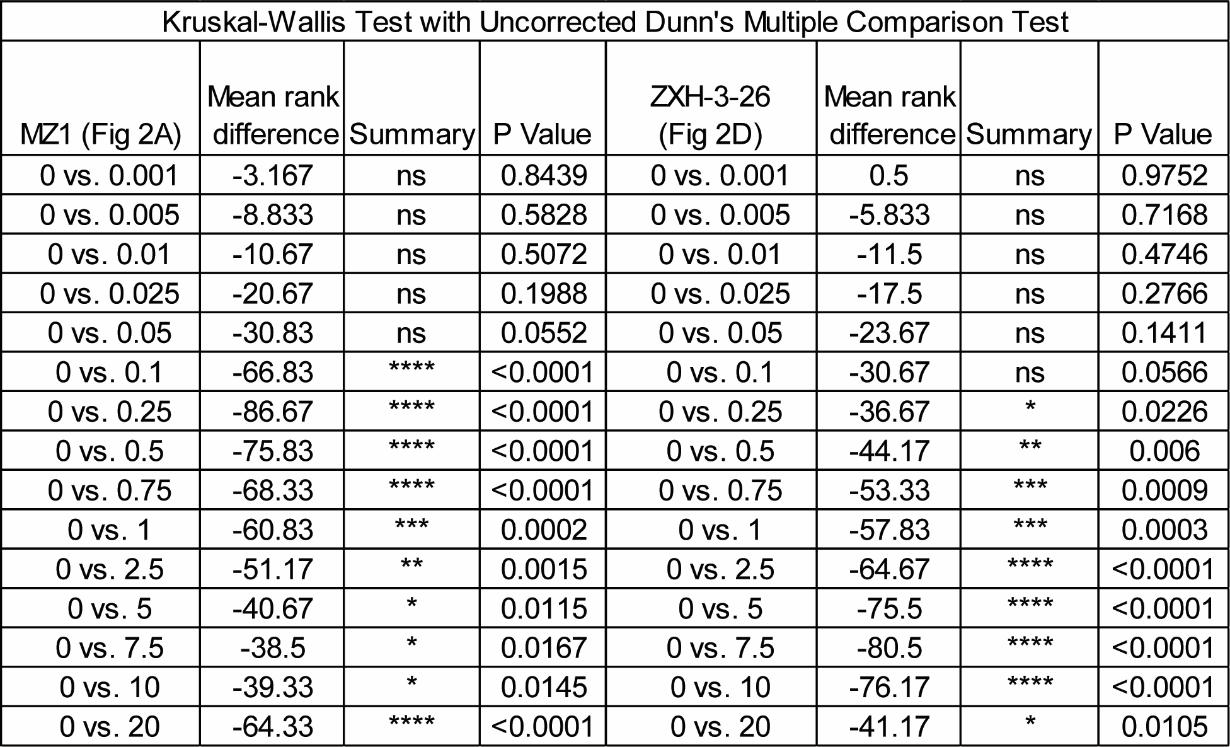


Table S1 – Statistical analysis of 16-point dose response curve for MZ1 and ZXH-3-26 from Figure 2A and 2D. All µM concentrations in columns 1 and 5 are compared to the DMSO control (0).
